# Who self-medicates? Results from structural equation modeling in the Greater Paris area, France

**DOI:** 10.1371/journal.pone.0208632

**Published:** 2018-12-17

**Authors:** A. Vanhaesebrouck, C. Vuillermoz, S. Robert, I. Parizot, P. Chauvin

**Affiliations:** 1 Department of Social Epidemiology, Institut Pierre Louis d’Epidémiologie et de Santé Publique (UMRS 1136), INSERM, Sorbonne Université, Paris, France; 2 Research team on social inequalities, Centre Maurice Halbwachs (UMR 8097), CNRS, EHESS, ENS, Paris, France; 3 Department of General Practice, Sorbonne Université, Paris, France; B P Koirala Institute of Health Sciences, NEPAL

## Abstract

**Objectives:**

Our study aimed to describe the prevalence of self-medication among the Paris adult population and to identify the factors associated with self-medication.

**Materials and methods:**

This cross-sectional study was based on data collected from the SIRS cohort (a French acronym for “Health, inequalities and social ruptures”) in 2005 in the Paris metropolitan area using a face-to-face administration questionnaire among a representative sample of 3,023 French-speaking adults. Structural equation models were used to investigate the factors associated with self-medication in the overall population and according to income.

**Results:**

The prevalence of self-medication in the past four weeks was 53.5% in the Paris metropolitan area. Seven factors were directly associated with self-medication in the structural equation model. Self-medication was found more common among women, young people, in active employment or student, with a high income, but also among people with a health information seeking behavior, with a high daily mobility, and/or with a history of unmet healthcare needs due to economic reasons. When looking at these coefficients according to income, the association between self-medication and daily mobility appeared stronger in the bottom quartile of income whereas it was no longer significant in the rest of the survey population.

**Conclusion:**

Self-medication is a frequent practice in the Paris metropolitan area. This study confirms the role of some factors found to be associated with self-medication in the literature such as age or gender and draws attention to other factors rarely explored such as daily mobility, especially among people with a low income, or health information seeking behavior.

## Introduction

Self-medication has become a public health issue and involves stakeholders with divergent interests. In a context of increasing control of public spending, development self-medication is encouraged by public authorities [1,2]. It is a source of public savings because over-the-counter medicines are not reimbursed and avoid medical consultations which would also be reimbursed [2,3]. The costs of healthcare are thus transferred from the public finances (in France, Social security) to the households. At the same time, self-medication is a source of profit for the pharmaceutical industry and drugstores because this transfer of costs goes with a deregulation of the prices of medicines. In spite of pharmacists being given a greater role in providing advice, iatrogenic risks related to self-medication lead a part of the medical community to claim for more regulation of self-medication [4–6]. Medicines may be tricky to handle for patients because of their plurality–the plurality of their dosages and the risk of drug interaction [7]. Self-medication may also mask the symptoms of a serious disease, leading to a delay in diagnosis. Attitudes towards self-medication vary somewhat among the patients [8]. Some patients are opposed to self-medication, because they are afraid to take medicines beyond control of physicians or argue that medicines should be reimbursed. Others, however, support self-management of care and argue that self-medication frees them from dependence of physicians and avoid the wait time before a consultation [8].

Self-medication covers different products and situations and its definition vary according to studies and to institutions, making it difficult to compare self-medication measures. In 1998, the World Health Organisation (WHO) defined self-medication as “the selection and use of medicines by individuals to treat self-recognized illnesses or symptoms”, including herbal and traditional products [9]. This is a broad definition: self-medication may here involve purchase of medicines, reuse of medicines kept at home or medicines provided by relatives and includes medicines non-recognized by conventional medicine.

In the medical literature, most of the studies dealing with self-medication target conventional drugs with marketing authorization, some consider drugstore products, dietary supplements or some foods. In France, the official definition states that self-medication is restricted to the delivering, in a pharmacy, of non-prescribed optional prescription drugs [1] (in France, drugs with a marketing authorization are available only in pharmacies). Under that definition, the French self-medication market accounted for 10.4% of turnover from ambulatory sale of drugs in 2015 [10]. Nevertheless, this definition appears too restrictive to capture self-medication related behaviors. Notably, medicines purchased are not necessarily used and we think that reuse of medicines kept at home or provided by relatives should enter into the field of self-medication, even in the case of prescribed drugs.

In our study, participants were interviewed on behaviors related to self-medication. As the determinants of self-medication are plentiful and multidimensional, we used structural equation modeling. The objective of our study was threefold: to measure the frequency of self-medication among French speaking adults in the Greater Paris area; to identify sociodemographic factors and behaviors associated with self-medication; to compare factors associated with self-medication according to income.

## Material and methods

### Participants and settings

The SIRS cohort (a French acronym for “Health, inequalities and social ruptures”) is a representative socio-epidemiological survey of the French-speaking adult population in the Paris metropolitan area [11]. The Paris metropolitan area is 814 square kilometers and had 6.3 million inhabitants by the 1999 census. The survey employed a stratified, 3-level random procedure. In the first step, fifty census blocks with approximately 2000 inhabitants each were selected, over-representing the poorest neighborhoods. In the second step, sixty households were randomly selected from each surveyed census block. In the final step, one adult was chosen from each household by the birthday method.

Data was collected during three waves in 2005, 2007 and 2010. Analyses are based on data collected in 2005 (from 1 September to 1 December) because the respondents were not asked for self-medication in the next waves. Twenty-nine percent of the people contacted declined to answer the survey and 5% were excluded because they did not speak French (3%) or because they were too sick to answer the questions (2%). A questionnaire was completed by investigators during home visits to a random sample of 3,023 people. More details on the methodology of the SIRS study can be found elsewhere [12,13].

This study received legal authorization from two French national authorities for non-biomedical research: the *Comité consultatif sur le traitement de l’information en matière de recherche dans le domaine de la santé* (CCTIRS) and *the Commission nationale de l’informatique et des libertés* (CNIL) [14]. The participants provided their verbal informed consent. Written consent was not necessary because this survey did not fall into the category of biomedical research (as defined by French law).

### Variables of interest

#### Outcome

In this study self-medication is defined by the answer “yes” to the following question: “Did you consume at least once a medicine without advice from a doctor over the past 4 weeks?” The following sentences could be used to relaunch twice the respondents who gave a negative answer: “We are talking about any medicines sold in a pharmacy, with or without prescription” and “Not even aspirin or pain killers? For women: not even a morning after pill?”. The definition used in this study includes purchase, reuse and sharing of medicine–the meaning of the word medicine being left to the discretion of the respondents.

#### Choice of covariates

In this paper, covariates were chosen on the basis of a preliminary work on the SIRS cohort [15] and analysis of literature. Analysis of literature was restricted to European studies on general population and on a wide range of drugs, published after 2000, in order to preserve the comparability of the results. Self-medication was found more frequent for women [2,16–19], for young [16,18,20,21] or working age people [2] and for people with a high level of education [2,16–21] in most of the studies. It was found more frequent as well for people with a high income [2], consumption of alcohol [16], smoking habit [16], social support and daily mobility [22]. For the self-perceived health and chronic disease factors, the association with self-medication was positive [16,17], not significant [2,18] or negative [16,19] according to the study.

#### Construction of latent variables

Structural Equation Modeling (SEM) is a generic analytical tool which use is common in behavioral and cognitive sciences [23] and extends to others disciplines [24,25]. The variables used in SEM framework can be observed variables or constructed variables (not observed), called constructs or latent variables [26]. SEM allows the modeling of associations between the covariates. The graphical construction is called path diagram and is made of boxes and ellipses linked via arrows. Observed variables are represented by a box, and latent variables by a circle or an ellipse [27]. An individual path (arrow) in SEM is tested in the same way the regression coefficient is in regression analysis [28].

The 10 following latent variables were assumed to be associated with self-medication:

1. Socioeconomic status was constructed from three indicators: the level of education (none or primary/secondary/post-secondary), the monthly household income per consumption unit (unweighted quartiles) and the professional status (employed or student versus others). Consumption units are a weighting scheme for households’ members defined by the Organisation for Economic Co-operation and Development.
2. Social support was constructed from four indicators: to have someone you can count on (yes/no), the feeling of being surrounded (very surrounded or quite surrounded versus quite alone or very alone), the household size (1 versus 2 or more) and the number of friends (at least one versus none).
3. Health information seeking was constructed from six indicators. The respondents were asked if they had searched for information or advice outside a medical consultation in the three past years (yes/no) on the following topics: an illness or a symptom, a medicine or a treatment, healthcare, alternative medicines or traditional remedies, diets and lastly on depression, anxiety, stress or mental health.
4. Daily mobility depicted the perimeter of daily activities and leisure around the housing. It was constructed from five indicators which specify the place where they did the following activities: going to a restaurant or café, meet with friends, go for a walk, go shopping and go to the post or to the bank (mainly outside the neighborhood/both within and outside the neighborhood/mainly inside the neighborhood or does not do this).
5. Mistrust in physicians was constructed from three indicators: thinking that the physicians best know what is good for ill people (no/yes), to have ever been victim or witness of a medical mistake (yes/no) and to have asked for a second medical opinion on its own initiative in the 12 last months (yes/no).
6. Chronic disease was constructed from three indicators: a chronic health condition (yes/no), a regular treatment or follow-up (yes/no) and at least one disease cited on a list in the 12 last months (yes/no, see note in Table 1).
7. Self-perceived health was constructed from three indicators: general health (very good/good versus fair/poor/very poor), physical health (ibid) and mental health (ibid).
8. Self-susceptibility to disease was based on the three following proposals: “becomes sick more easily than others” (agree/disagree), “the body does not seem to resist very well to disease” (agree/disagree) and “generally catches everything that lays around” (agree/disagree).
9. Disease prevention was constructed from three indicators: vaccination against tetanus up to date (yes/no or do not know), last visit to the dentist (≤1 year versus >1 year), to have ever been tested for HIV infection (yes/no or do not know).
10. Health risk behaviors was constructed from two indicators: daily smoking (yes/no) and potential alcohol abuse (at least one “yes” answer to the 4 CAGE questions [29] versus others).

**Table 1:** Characteristics of the population (weighted data, n = 3023).

	Distribution in the population		Prevalence of self-medication	
	n	%	%	p
Gender				
Women	1602	53.0	55.3	0.076
Men	1421	47.0	51.5	
Age (years)				
18–39	1329	44.0	61.7	<0.001
40–59	1030	34.1	53.0	
>60	664	22.0	38.0	
*Socioeconomic status*				
Education				
None/Primary	297	9.8	36.1	<0.001
Secondary	1174	38.9	49.7	
Post-secondary	1551	51.3	59.7	
Income (monthly household income per consumption unit, euros)				
<1000	650	21.5	45.2	<0.001
1000–1500	702	23.2	52.6	
1500–2200	797	26.4	55.1	
>2200	874	28.9	58.8	
Employed or student				
Yes	1919	63.5	60.0	<0.001
No	1101	36.5	42.0	
*Health information seeking (outside a medical consultation*, *in the past 3 years)*				
Researching information or advice on a medicine or a treatment				
Yes	1034	34.3	64.4	<0.001
No	1980	65.7	47.7	
Researching information or advice on an illness or a symptom				
Yes	596	19.8	62.5	<0.001
No	2410	80.2	51.0	
Researching information on healthcare news				
Yes	663	22.0	60.6	<0.001
No	2357	78.0	51.4	
*Self-perceived susceptibility to disease*				
Becomes sick more easily than others				
Agree	303	10.1	51.5	0.501
Disagree	2709	89.9	53.8	
The body does not seem to resist very well to disease				
Agree	377	12.6	51.2	0.388
Disagree	2623	87.4	53.9	
Generally catches everything that lays around				
Agree	562	18.7	60.7	0.001
Disagree	2445	81.3	51.9	
*Daily mobility (within or outside the neighborhood of residence (NR))*				
Where they usually go to the restaurant or to the café				
Mainly within the NR	991	32.8	46.7	<0.001
Both within and outside the NR	616	20.4	54.8	
Mainly outside the NR	1409	46.7	57.7	
Where they usually go for a walk				
Mainly within the NR	898	29.8	46.4	<0.001
Both within and outside the NR	1039	34.5	54.8	
Mainly outside the NR	1072	35.6	58.5	
Where they usually meet friends				
Mainly within the NR	645	21.5	45.8	<0.001
Both within and outside the NR	1134	37.7	52.9	
Mainly outside the NR	1228	40.8	58.0	
*Self-perceived health*				
General health				
Very good/Good	2335	77.3	56.1	<0.001
Very poor/Poor/Fair	686	22.7	44.8	
Physical health				
Very good/Good	2316	76.7	56.0	<0.001
Very poor/Poor/Fair	704	23.3	45.6	
Psychological health				
Very good/Good	2330	77.2	54.6	0.046
Very poor/Poor/Fair	690	22.8	49.7	
*Chronic disease*				
Regular treatment or follow-up				
Yes	1191	39.4	44.2	<0.001
No	1831	60.6	59.5	
Chronic health condition				
Yes	972	32.2	47.3	<0.001
No	2049	67.8	56.5	
At least one disease among those listed over the last 12 months^a^				
Yes	1695	56.1	51.9	0.101
No	1328	43.9	55.5	
*Social support*				
Someone you can count on				
Yes	2864	94.7	54.4	<0.001
No	159	5.3	37.5	
Household size (number of adults and children)				
1	573	18.9	48.6	0.012
2–3	1468	48.6	52.8	
>3	983	32.5	57.4	
*Mistrust in physicians*				
Physicians do not best know what is good for ill people				
Agree	339	11.2	58.0	0.119
Disagree	2678	88.8	52.9	
Victim or witness of a medical mistake (whole life)				
Yes	618	20.4	58.8	0.013
No	2404	79.6	52.2	
*Health risk behaviors*				
Potential alcohol abuse^b^				
Yes	465	15.4	61.8	0.001
No	2549	84.6	52	
Current daily smoking				
Yes	764	25.3	57.9	0.019
No	2258	74.7	52	
*Disease prevention*				
Dietary supplements, vitamins or minerals intake over the past 4 weeks				
Yes	697	23.1	66.3	<0.001
No	2321	76.9	49.7	
*Others*				
Unmet healthcare needs due to economic reasons in the 12 last months				
Yes	512	16.9	62.7	<0.001
No	2509	83.1	51.6	
Medical science is not effective for all health problems				
Agree	1483	49.3	57.5	<0.001
Disagree	1525	50.7	49.6	

^a^ Asthma, allergy, diabetes, cataract, high blood pressure, heart attack, stroke, chronic bronchitis, emphysema, rheumatoid arthritis, arthrosis, osteoporosis, gastric or duodenal ulcers, cancer, migraine, anxiety, depression, other disease

^b^ At least one “yes” response to the CAGE questionnaire

### Basic assumptions: Latent variables

1. We made the assumption that a higher socioeconomic status would be associated with more self-medication because people with a higher socioeconomic status can afford non-reimbursed medicines. Moreover, they have on average a better access to information tools such as internet [13,30] and a higher understanding of medication to deal with it [31], and have a higher social support [32,33].
2. A higher social support was assumed to be associated with more self-medication because more relatives may share medicines and advice on medicines.
3. Health information seeking was assumed to be associated with more self-medication because this practice is indicative of a self-management of health [34] and provide useful information for the self-diagnosis of illnesses and self-medication.
4. A higher daily mobility was assumed to be associated with more self-medication because it enables to get close to cheap pharmacies, given that pharmacies can freely set prices of OTC medicines [1]. Moreover, by analogy with other types of health care activities [35], a higher daily mobility may result in a higher diversity of social interaction opportunities, which may contribute to modifying health-care norms [36] and notably self-medication.
5. Mistrust in physicians was assumed to be associated with more self-medication, on the basis of a qualitative study suggesting that self-medication was for some people an avoidance strategy of physicians, often as a result of disappointed experiences with physicians [37].
6. Suffering from a chronic disease was assumed to be associated with less self-medication because in this situation people are more likely to benefit from sustained medical care. Their doctor prescribes the medicines they need and warn them of dangers of self-medicating, notably medicine interactions. Also, people suffering from chronic diseases often have a regular intake of several medicines [38] so they may not want to take one more.
7. A better self-perceived health was assumed to be associated with more self-medication. Chronic disease is more common for people with a poor perceived health [39] and is assumed to be associated with less self-medication.
8. Self-perceived susceptibility to disease was assumed to be associated with more self-medication because when people do not have confidence in their own abilities to prevent or fight against disease they are more likely to seek for outside assistance. In this situation self-medication is a form of assistance.
9. Disease prevention was assumed to be associated with more self-medication because in both cases people are more likely to have a great concern about their health.
10. Health risk behaviors was assumed to be associated with self-medication on the basis of the literature [16].

### Basic assumptions: Other factors

1. Younger people were assumed to self-medicate more often because they generally have a better command of health information tools, a higher daily mobility [35] and a higher social support [40]. Moreover, they grew up during a period when medical authority was questioned by society [41] so we assumed that they are more likely than older people to take medicines without prescription.
2. Women were assumed to self-medicate more because of a higher social support [42] and a greater concern about self-care than men [43].
3. To believe that medicine is not effective for all health problems was assumed to be associated with less self-medication because in this situation sick individuals are more likely to use other healthcare resources than medicines.
4. We made the assumption that a history of unmet healthcare needs due to economic reasons in the last 12 months was found to be associated with more self-medication, in accordance with results from previous analysis on the SIRS cohort [15]. This could be because people with a history of unmet healthcare needs are more likely to have no complementary health insurance [44]. All the French people benefit from a public health insurance which partly pay the majority of medical care and may subscribe to a private complementary health insurance. For people without a complementary health insurance and who choose to take a treatment anyway, self-medication is often cheaper than a medical consultation, particularly when it avoids excess from medical consultations and additional investigations. Self-medication even costs nothing for people who reuse medicines kept at home.

### Statistical methods

All descriptive data in this article were weighted to account for the complex sample design (notably, the design effect associated with cluster sampling and the overrepresentation of poorer neighborhood) and for the post-stratification adjustment for age and sex according to the general census of the population. For each observed variable, weighted proportions of self-medication were compared using Pearson chi-squared tests. Weighted descriptive data and univariate analysis were compute with the package “survey” in R software (R version 3.2.5).

Latent variables were assessed in a three-step approach. In the first step, we examined the correlation matrix between observed variables. Within each latent variable, an observed variable was retained if it was associated with each of the other observed variables with a Pearson correlation coefficient >0.3. As a latent variable must include at least two observed variables, it was dropped if there was only one observed variable left. In the second step, the unidimensionality of the latent variables was assessed using scree plots. In the third step, we estimated measurement models using confirmatory factor analysis (CFA) and finally, tested a structural equation model.

Models were estimated with the WLSMV estimator (Weighted Least Squares estimation with robust standard errors and a Mean and Variance adjusted test statistic) because of the use of binary and ordinal variables. Given that this estimator is for complete data only, respondents with missing data for any factor included in the model were excluded from the model. In all CFA and structural equation models, goodness of fit was assessed with robust criteria in order to take the non-normal distribution of the data into account. Two criteria were used: the robust comparative fit index (CFI) and the robust root mean square error of approximation (RMSEA). The CFI ranges from 0 to 1, with values above 0.90 corresponding to an acceptable fit [45], while an RMSEA value below 0.08 is recommended [46]. For an easier interpretation of the coefficients of the models, standardized estimates were reported. Standardized estimates (ranging from -1 for a complete negative association to 1 for a complete positive association) can be interpreted with reference to other parameters in the model, and relative strength of associations can be compared. Moreover, observed variables from dropped latent variables were allowed to be entered in the hypothetical model, especially if they were found to be associated with self-medication in the literature.

To improve goodness of fit, new relationships can be added following the residual correlation matrix (i.e. the difference between the model implied-correlations matrix and the corresponding correlations in the data) or non-significant relationships (p value <0.05) can be removed. Factors no longer directly or indirectly associated with self-medication can be removed from the model. If the goodness-of-fit indices of the model remain unsatisfactory, relationships with a standardized coefficient _std_β≤0.05 can be deleted.

The resulting model was firstly described for the whole sample, and then compared between two subgroups of people: those belonging to the bottom quartile of income versus the others.

CFA and structural equation modeling were performed with the “lavaan” package in R software.

## Results

### Description of the survey population

Characteristics of the survey population are presented in the Table 1. The French-speaking adult population of the Paris metropolitan area had quite a high level of education (51.3% received post-secondary education) and was quite young (44% were between 18 and 39). Sixty per cent of them were employed or a student. Almost all respondents mentioned if they self-medicated in the past 4 weeks (3020/3023, 99.9%). A history of self-medication was found for 53.5% of the survey population (_95%_CI [51.4–56.0]). Twenty-four of the 29 variables presented in the Table 1 were associated with self-medication (p<0.05) in univariate analysis.

### Assessment of latent variables

Among the ten latent variables assessed, four were dropped because of lack of correlation between observed variables: disease prevention, health risk behaviors, mistrust in physicians and social support. For the same reason, some observed variables were removed from “socioeconomic status”, “daily mobility” and “health information seeking” latent variables. All of the six remaining latent variables were unidimensional (S1 Fig) and were entered into a CFA model. The “socioeconomic status” latent variable was removed from the CFA model to obtain satisfactory goodness of fit.

The five remaining latent variables were: health information seeking, chronic disease, self-perceived susceptibility to disease, daily mobility and self-perceived health (S2 Fig). The model had good fit: the robust CFI was 0.973 and the robust RMSEA was 0.030 CI [0.026–0.034]. All indicators of all latent variables had a p value <0.001.

### SEM of self-medication

**Fig 1:**
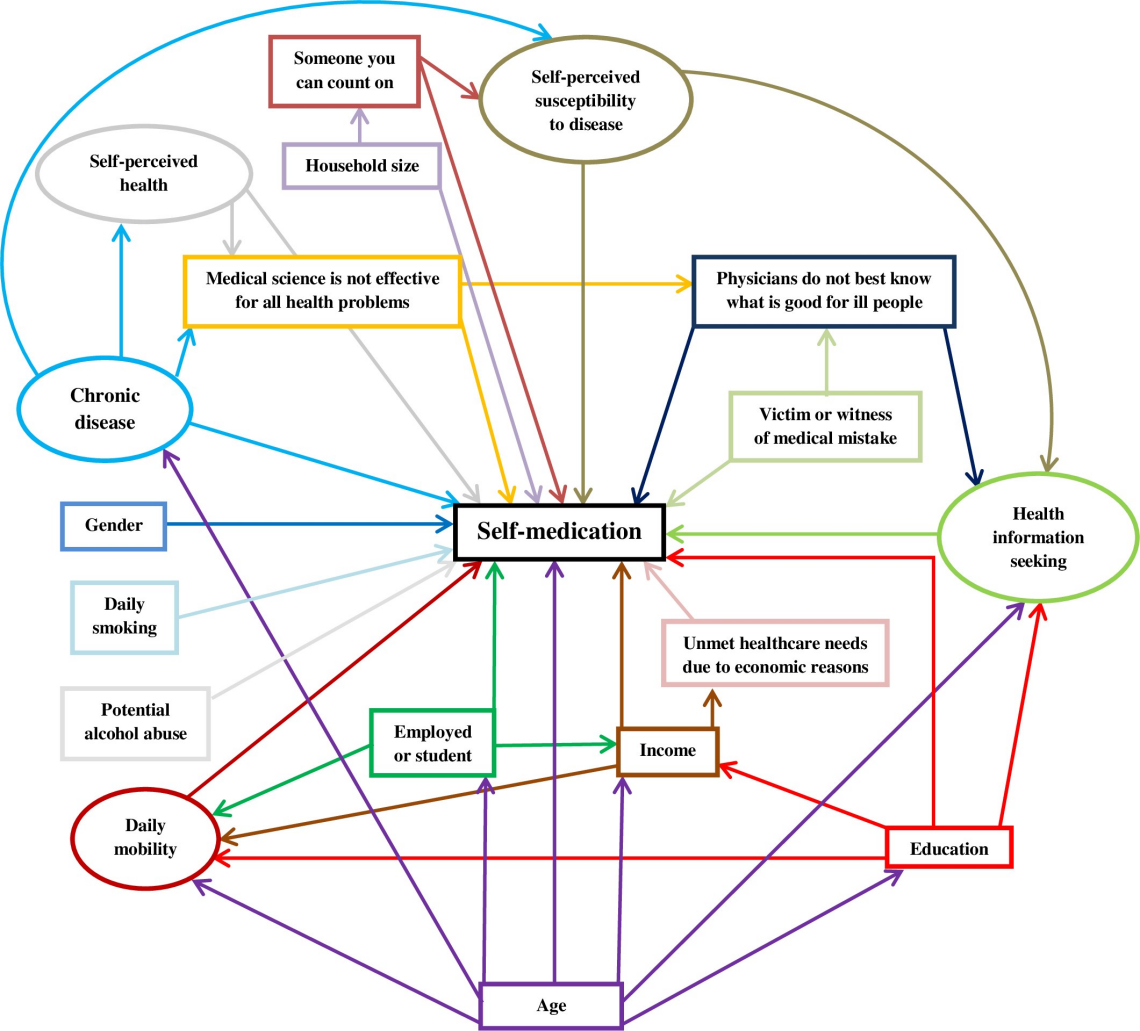
Hypothesized path diagram of self-medication, n = 2872. Ellipses: latent variables; boxes: observed variables. Arrows starting from the same explanatory variable are in the same color. For the sake of clarity, indicators of latent variables are not shown. Indicators of *self-perceived health* are *general health*, *physical health* and *psychological health*. Indicators of *chronic disease* are *regular treatment or follow-up*, *chronic health condition* and “*at least one condition among those listed over the last 12 months”* (see note in Table 1). Indicators of *daily mobility* are “*where they usually meet friends”*, *“where they usually go for a walk”* and *“where they usually go to the restaurant*, *to the café”*. Indicators of *health seeking information* are re*searching information or advice on an illness or a symptom*, re*searching information on healthcare news* and re*searching information or advice on a medicine or a treatment*. Indicators of *self-perceived susceptibility to disease* are “You *generally catch everything that lays around”*, *“You become ill more easily than others”* and *“Your body do not seem to resist very well to disease”*.

The hypothetical structural equation model is shown in Fig 1. Two latent variables (chronic disease and self-perceived health) and four observed variables (victim or witness of a medical mistake, household size, dietary complements, vitamins or minerals intake and current daily smoking) were removed from the model because they were not significantly associated with self-medication. Two relationships were added in the model: self-perceived susceptibility to disease was found to be influenced by income and gender. After deletion of links with a standardized coefficient below 0.05, “someone you can count” on and “potential alcohol abuse” were removed from the model.

**Fig 2:**
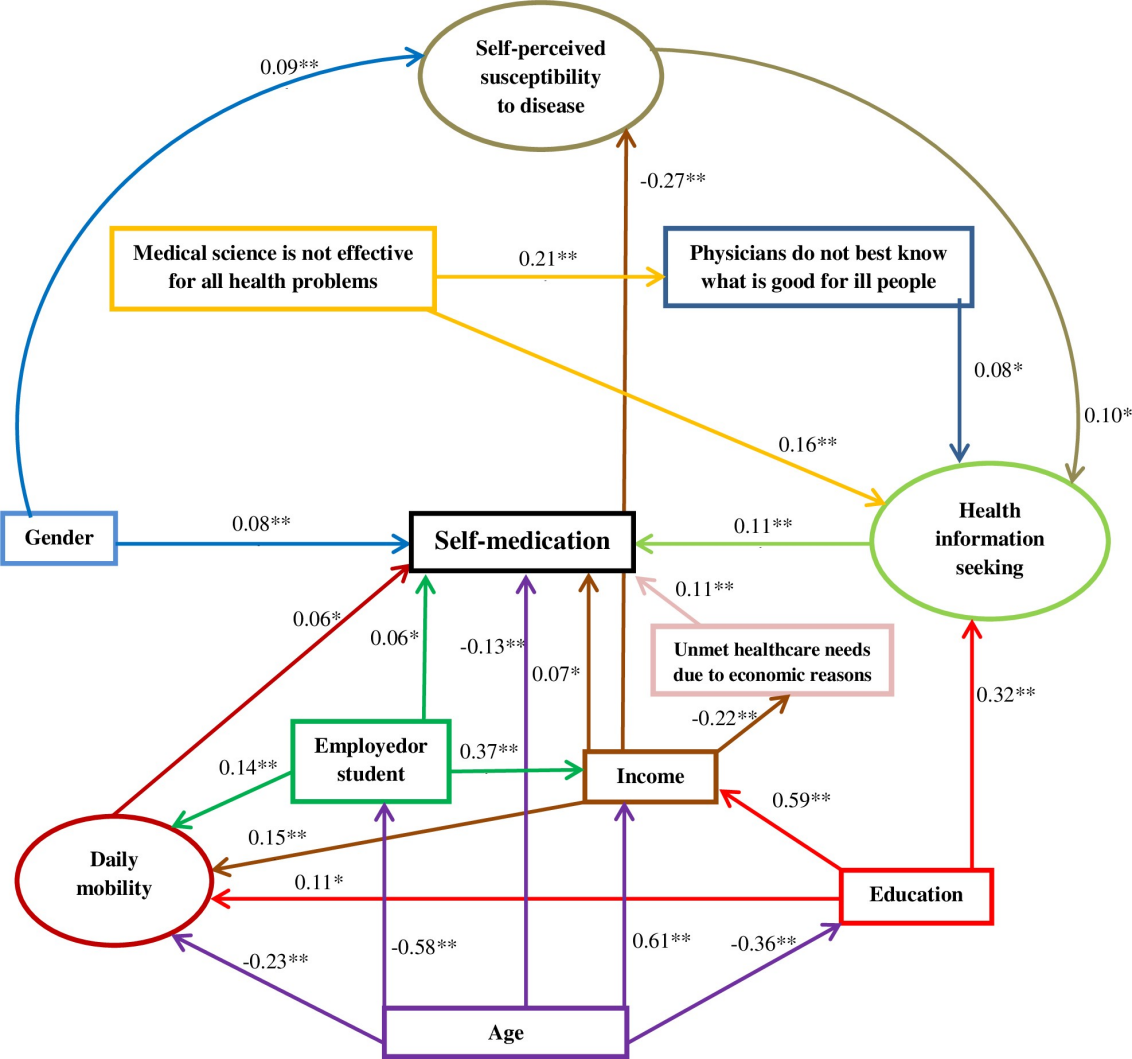
Path diagram of self-medication, n = 2893. Ellipses: latent variables; boxes: observed variables. Arrows starting from the same explanatory variable are in the same color. All coefficients are standardized. Robust CFI = 0.915. Robust RMSEA = 0.039 [0.036–0.042] *: <0.05 **: <0.001.

The final structural equation model is shown in Fig 2. One hundred and thirty respondents (4.3%) were excluded from the final model because of missing data. Two latent variables were directly associated with self-medication: self-medication was more frequent for people with a health information seeking behavior (_std_β = 0.11, p<0.001) and for people with high daily mobility (_std_β = 0.06, p = 0.020). Health information seeking was positively influenced by a high level of education (_std_β = 0.32, p<0.001), believing that “medical science is not effective for all health problems” (_std_β = 0.16, p<0.001), a high self-perceived susceptibility to disease (_std_β = 0.10, p = 0.001) and believing that “physicians do not best know what is good for ill people” (_std_β = 0.08, p = 0.002). Daily mobility was positively influenced by income (_std_β = 0.15, p<0.001), being employed or student (_std_β = 0.14, p<0.001) and a high level of education (_std_β = 0.11, p<0.001) and tended to be lower for older people (_std_β = -0.23, p<0.001). Five observed variables were directly associated with self-medication: self-medication appeared to be more frequent for young people (_std_β = -0.13, p<0.001), for those with a history of unmet healthcare needs due to economic reasons (_std_β = 0.11, p<0.001), for women (_std_β = 0.08, p<0.001), for those with a high income (_std_β = 0.07, p = 0.001) and for people employed or student (_std_β = 0.06, p = 0.014).

Although a higher income was directly associated with more self-medication, it was indirectly associated with less self-medication: a higher income was associated with less renouncement to healthcare for economic reasons (_std_β = -0.22, p<0.001) and self-medication was more common for people with a history of unmet healthcare needs due to economic reasons (_std_β = 0.11, p<0.001). The goodness-of-fit indices of the model were satisfactory (robust CFI = 0.915 and robust RMSEA = 0.039 [0.036–0.042]).

Among people within the bottom quartile of income, 45.2% (_95%_CI [40.9–49.6]) self-medicated. Path diagrams and coefficients of the structural equation models for the bottom quartile of income and for the top three quartiles of income are in annex (S3 and S4 Figs). The structure of these paths diagrams is similar to the one presented in Fig 2, except for the observed variable income and its related relationships which were removed. Self-medication was directly associated with daily mobility in the bottom quartile of income (_std_β = 0.17, p = 0.001) but not in the top three quartiles of income (_std_β = 0.02, p = 0.505). The influence of education on daily mobility was stronger in the bottom quartile of income (_std_β = 0.31, p<0.001) than in the top three quartiles (_std_β = 0.07, p = 0.042). Moreover, the relationship between age and being employed or student was weaker in the bottom quartile of income (_std_β = -0.36, p<0.001) than in the top three quartiles (_std_β = -0.67, p<0.001). The other relationships remained quite stable according to income (Δ_std_β≤0.05 for those which directly involve self-medication).

## Discussion

Half of the participants (53.5%) took medicine without medical advice at least once in the four weeks before the interview. Seven factors were found to be associated with self-medication in the SEM model: age, gender, income, professional status, health information seeking, daily mobility and a history of unmet healthcare needs due to economic reasons. The strength of these associations was of the same order of magnitude. According to income, the association between daily mobility and self-medication was found only among the people in the bottom quartile of income and was stronger for this population.

Self-medication was a common practice in the Greater Paris area in 2005, as more than half of the participants self-medicated during the four weeks before the interview. The four weeks reporting period probably missed a large part of the population with an occasional use of medicines, suggesting that our results most likely refer to a regular use of medicines rather than an occasional use. The frequency of self-medication cannot be extrapolated to France, as the Greater Paris area is not representative of the French population. The Greater Paris area is more urbanized, richer and is better endowed with medical services than the rest of France [47]. Furthermore, the frequency of self-medication can hardly be compared with other studies because of sources of heterogeneity in terms of season of the year, the definition used for self-medication and the reporting period.

Many factors were found to be associated with self-medication in the SEM model. The strength of these associations was of the same order of magnitude, suggesting that no factor is decisive for predicting self-medication. Our results are consistent with the literature for gender [2,16–19] and age [2,16,18,20,21]. Self-medication was found more frequent for women and for young people. Moreover, the association between age and self-medication was found partly mediated by daily mobility, thus confirming our initial hypothesis.

Five out of the ten latent variables assumed to be associated with self-medication were finally assessed in the SEM model. Three of them were found to be associated with self-medication: health information seeking, daily mobility and self-perceived vulnerability to disease.

A positive association was found between health information seeking and self-medication, in accordance with our assumption that these practices are both indicative of self-management of care [34,48]. The positive association between self-medication and health information seeking suggests that patients do not have an indiscriminate use of medicines as some physicians fear. However, this is mitigated by the fact that we did not take into account the source, the quality and the understanding of sought information (particularly on the internet) in our study [49]. Health information can come as much from health professionals as from relatives, discussion forums, non-specialized magazines or advertising (only allowed for OTC medicines in France).

The positive association found between daily mobility and self-medication is also in accordance with our assumptions that daily mobility enables to get close to cheap pharmacies and may contribute to modifying health-care norms [36]. A positive association was found between self-perceived susceptibility to disease and self-medication, in accordance with our assumption that people with a higher self-perceived susceptibility to disease are more likely to seek for outside assistance. This association was mediated by health information seeking. This mediation can be explained by the fact that health information is a kind of assistance that reassures people with a high self-perceived susceptibility to disease, notably in the case of common symptoms, and bring them tools to self-medicate.

Suffering from a chronic disease was not associated with self-medication in our study. In the literature, the association with self-medication was positive [17], not significant [18] or negative [16,19] according to the study. We may be in the presence of opposite influences (as for the income discussed below). Some people with a chronic disease may less self-medicate because of a regular medical follow-up and/or a stringent treatment, as we previously assumed, while other people may self-medicate more, because they have become more self-reliant owing to experience accumulated on self-management of health.

The SEM model highlighted a complex association between income and self-medication. The income was associated with self-medication both directly and indirectly, mediated by “a history of unmet healthcare needs due to economic reasons”. The direct association was positive, which is in accordance with our initial assumption that people with a higher socioeconomic status can afford non-reimbursed medicine. Nevertheless, the association between income and a history of unmet healthcare needs was negative, and the association between a history of unmet healthcare needs and self-medication was positive, resulting in a negative indirect association between income and self-medication. The positive association between a history of unmet healthcare needs and self-medication is in accordance with the assumption by knowing that people with unmet healthcare needs are more likely to have no complementary health insurance [44], self-medication avoid excess from medical consultations and complementary investigations.

No direct association between level of education and self-medication was found in the SEM model. The effect of the level of education on self-medication was mediated by three factors: health information seeking, the income and daily mobility. The absence of residual direct association between level of education and self-medication suggests that most of the effect of the level of education, a well-known determinant of self-medication [2,16–21], could be explained by these three factors.

The association between daily mobility and self-medication was inconsistent according to the income. This result can be explained in the light of the relationship between self-medication and the income. In one hand, self-medication was less common among the poorest people. This probably because some OTC medicines are too expansive for them. One the other hand, self-medication is more common for those with a high daily mobility only among the poorest people. This can be explained by a better access to cheap pharmacies. Indeed, prices of over-the-counter medicines may vary significantly according to the pharmacy as they are free to set their prices for OTC medicines. A price recording study of 13 common health products was conducted in 76 pharmacies in 36 French departments in 2010. Prices ratios and prices differences between the cheapest and the more expensive pharmacies were found to range respectively from 1.5 to 3.3 and from 2.1 to 13.9 euros according to the product [50]. A high daily mobility, which appears to facilitate access to OTC medicines among the poorest people, would have no impact on the people with sufficient income to be insensitive to variations of prices. The association between self-medication and daily mobility would then reveal the difficulties in accessing self-medication for the poorest people. This association can thus be understood as an indicator of health inequalities.

The association between the level of education and daily mobility was also inconsistent according to the income, being stronger for the bottom quartile of income than for the three upper quartiles of income. Apart from these few differences mentioned above, the relationships identified in the SEM model were consistent according to the income, which is indicative of a good internal consistency.

One strength of our study is the wealth of the SIRS cohort data. Many behavioral information was available, which allowed us to build latent variables and to assess the relationship between self-medication and factors that have been little explored so far. Another strength is that bias usually encountered in questionnaire-based surveys are limited in our study. Firstly, the non-response rate (29%) is below average for a face-to-face questionnaire in population-based surveys [51–53]. Secondly, the exclusion criteria were met only for 5% of eligible participants, thus limiting selection bias. Thirdly, misclassification of self-medication status due to declarative data is probably low, as the risk of underreporting of self-medication was reduced by the relaunch instructions given to the interviewers and a short reporting period. Furthermore, the desirability bias is probably marginal. Though some people may be reluctant to report self-medication to some physicians, the participants were interviewed by non-medical trained interviewers. The age of the data may however alter the importance of some of the findings, which must be confirmed by other studies.

## Conclusion

Half of the Greater Paris area’s population self-medicate in a context where self-medication involves various actors with divergent positions who globally encourage the development of self-medication. Our study draws attention to factors associated with self-medication that have been little explored to date and which may interest physicians and pharmacists, as daily mobility, notably among the poorest people, and health information seeking. Inequalities in access to health information and uneven quality of health information call public authorities and health professionals to train people to self-medicate thoughtfully. Indeed, improving health literacy in communities is essential to reduce health risks related to self-medication. The lower frequency of self-medication found for the poorest people and for those with a low level of education are indicative of social inequalities in access and/or recourse to self-medication that need to be addressed, especially if the development of self-medication is expected to continue.

## Supporting information

S1 Appendix Questionnaire of the SIRS cohort of the year 2005. (PDF)

S1 Fig Scree plots: Assessment of the unidimensionality of the latent variables. (PDF)

S2 Fig Results of confirmatory factor analysis. **N = 2918.** Ellipses: latent variables; boxes: observed variables. All coefficients are standardized and have a p value <0.001. Robust CFI = 0.973. Robust RMSEA = 0.030.
(PDF)

S3 Fig Path diagram of self-medication, in the bottom quartile of household income, n = 707. Arrows starting from the same explanatory variable are in the same colour. Ellipses: latent variables; boxes: observed variables. All coefficients are standardized. Robust CFI = 0.882. Robust RMSEA = 0.048.
(PDF)

S4 Fig Path diagram of self-medication, in the top three quartiles of household income, n = 2186. Arrows starting from the same explanatory variable are in the same colour. Ellipses: latent variables; boxes: observed variables. All coefficients are standardized. Robust CFI = 0.946. Robust RMSEA = 0.030.
(PDF)
