# Supplementary material for: Who self-medicates? Results from structural equation modeling in the Greater Paris area, France
Source: PLoS One. 2018 Dec 17;13(12):e0208632. doi: 10.1371/journal.pone.0208632 (PMC6296538; doi:10.1371/journal.pone.0208632)
Supplement: S1 Appendix — (PDF) [file pone.0208632.s001.pdf]

Inserm
Institut national
de la santé et de la recherche médicale

CNRS
CENTRE NATIONAL
DE LA RECHERCHE
SCIENTIFIQUE

Enquête SIRS en Ile-de-France

2005

NumId

Quartier		Adresse			Personne	

Nom Enquêteur : .... N° Enq. :

Date : é é é é é é é é é é é é

A- FICHE MENAGE

Pouvez-vous énumérer toutes les personnes qui résident habituellement dans votre logement, en précisant leur prénom et quelques caractéristiques.

	1	2	3	4	5	6	7	8	9
**A1. Prénom**									
**A2. Sexe**<br>1. Homme 2. Femme	|___|	|___|	|___|	|___|	|___|	|___|	|___|	|___|	|___|
**A3. Lien avec la personne de référence ("chef de ménage")**<br>1. personne de référence (PR)<br>2. conjoint de PR<br>3. enfant de PR ou du conjoint<br>4. père/mère de PR ou du conjoint<br>5. autre parent de PR ou du conjoint<br>6. salarié logé ou domestique<br>7. ami, non apparenté	|___|	|___|	|___|	|___|	|___|	|___|	|___|	|___|	|___|
**A4. Age**<br>en années révolues	|___|___|	|___|___|	|___|___|	|___|___|	|___|___|	|___|___|	|___|___|	|___|___|	|___|___|
**A5. Niveau dõétudes**<br>1. jamais scolarisé<br>2. primaire<br>3. secondaire<br>4. supérieur	|___|	|___|	|___|	|___|	|___|	|___|	|___|	|___|	|___|
**A6. Occupation actuelle**<br>actif<br>1. exerce un emploi<br>2. chômeur<br>inactif<br>3. étudiant<br>4. retraité<br>5. au foyer<br>6. autre	|___|	|___|	|___|	|___|	|___|	|___|	|___|	|___|	|___|
**A7. Profession (actuelle ou dernière)**<br>Coder selon la grille jointe (Carte 1)	|___|	|___|	|___|	|___|	|___|	|___|	|___|	|___|	|___|
**A8. *Enquêteur* :**<br>1. médecin<br>2. infirmier<br>3. autre profession médicale	|___|	|___|	|___|	|___|	|___|	|___|	|___|	|___|	|___|

Enquêteur **:**

A9. Type de ménage : |___|
1. ménage dõune seule personne
2. ménage mononucléaire
3. ménage mononucléaire élargie
4. ménage pluri-nucléaire (plusieurs couples ou plusieurs « familles »)
5. ménage pluri-nucléaire élargi
6. famille monoparentale (élargie ou non)
7. ménage dõ« isolés »
8. autre

A10. Parmi toutes les personnes âgées de 18 ans ou plus, quelle est celle qui, la première, fêtera son anniversaire ?

N° dõidentification de la personne à interroger : |_____| ➔ **Complétez le NumId sur la couverture**

FICHE MENAGE - COMPLEMENTAIRE

	10	11	12	13	14	15	16	17	18
**A1. Prénom**									
**A2. Sexe**<br>1. Homme 2. Femme	|___|	|___|	|___|	|___|	|___|	|___|	|___|	|___|	|___|
**A3. Lien avec la personne de référence ("chef de ménage")**<br>1. personne de référence (PR)<br>2. conjoint de PR<br>3. enfant de PR ou du conjoint<br>4. père/mère de PR ou du conjoint<br>5. autre parent de PR ou du conjoint<br>6. salarié logé ou domestique<br>7. ami, non apparenté	|___|	|___|	|___|	|___|	|___|	|___|	|___|	|___|	|___|
**A4. Age**<br>en années révolues	|___|___|	|___|___|	|___|___|	|___|___|	|___|___|	|___|___|	|___|___|	|___|___|	|___|___|
**A5. Niveau dõétudes**<br>1. jamais scolarisé<br>2. primaire<br>3. secondaire<br>4. supérieur	|___|	|___|	|___|	|___|	|___|	|___|	|___|	|___|	|___|
**A6. Occupation actuelle**<br>actif<br>1. exerce un emploi<br>2. chômeur<br>inactif<br>3. étudiant<br>4. retraité<br>5. au foyer<br>6. autre	|___|	|___|	|___|	|___|	|___|	|___|	|___|	|___|	|___|
**A7. Profession (actuelle ou dernière)**<br>Coder selon la grille jointe (Carte 1)	|___|	|___|	|___|	|___|	|___|	|___|	|___|	|___|	|___|
A8. Enquêteur :<br>1. médecin<br>2. infirmier<br>3. autre profession médicale	|___|	|___|	|___|	|___|	|___|	|___|	|___|	|___|	|___|

Enquêteur **:**

A9. Type de ménage : |___|

1. ménage dõune seule personne
2. ménage mononucléaire
3. ménage mononucléaire élargie
4. ménage pluri-nucléaire (plusieurs couples ou plusieurs « familles »)
5. ménage pluri-nucléaire élargi
6. famille monoparentale (élargie ou non)
7. ménage dô« isolés »
8. autre

A10. Parmi toutes les personnes âgées de 18 ans ou plus, quelle est celle qui, la première, fêtera son anniversaire ?

N° dõidentification de la personne à interroger : |_____| ➔ **Complétez le NumId sur la couverture**

FICHE MENAGE ï COMPLEMENTAIRE

	19	20	21	22	23	24	25	26	27
**A1. Prénom**									
**A2. Sexe**<br>1. Homme 2. Femme	|___|	|___|	|___|	|___|	|___|	|___|	|___|	|___|	|___|
**A3. Lien avec la personne de référence ("chef de ménage")**<br>1. personne de référence (PR)<br>2. conjoint de PR<br>3. enfant de PR ou du conjoint<br>4. père/mère de PR ou du conjoint<br>5. autre parent de PR ou du conjoint<br>6. salarié logé ou domestique<br>7. ami, non apparenté	|___|	|___|	|___|	|___|	|___|	|___|	|___|	|___|	|___|
**A4. Age**<br>en années révolues	|___|___|	|___|___|	|___|___|	|___|___|	|___|___|	|___|___|	|___|___|	|___|___|	|___|___|
**A5. Niveau dőétudes**<br>1. jamais scolarisé<br>2. primaire<br>3. secondaire<br>4. supérieur	|___|	|___|	|___|	|___|	|___|	|___|	|___|	|___|	|___|
**A6. Occupation actuelle**<br>actif<br>1. exerce un emploi<br>2. chômeur<br>inactif<br>3. étudiant<br>4. retraité<br>5. au foyer<br>6. autre	|___|	|___|	|___|	|___|	|___|	|___|	|___|	|___|	|___|
**A7. Profession (actuelle ou dernière)**<br>Coder selon la grille jointe (Carte 1)	|___|	|___|	|___|	|___|	|___|	|___|	|___|	|___|	|___|
A8. Enquêteur :<br>1. médecin<br>2. infirmier<br>3. autre profession médicale	|___|	|___|	|___|	|___|	|___|	|___|	|___|	|___|	|___|

Enquêteur **:**

A9. Type de ménage : |___|
1. ménage dőune seule personne
2. ménage mononucléaire
3. ménage mononucléaire élargie
4. ménage pluri-nucléaire (plusieurs couples ou plusieurs « familles »)
5. ménage pluri-nucléaire élargi
6. famille monoparentale (élargie ou non)
7. ménage dő« isolés »
8. autre

A10. Parmi toutes les personnes âgées de 18 ans ou plus, quelle est celle qui, la première, fêtera son anniversaire ?

N° dőidentification de la personne à interroger : |_____| ➔ **Complétez le NumId sur la couverture**

B- RAPPEL

B1. Sexe

Homme	1
Femme	2

B2. Age

|_____| ans

C- LOGEMENT ET QUARTIER

« Pour commencer cette enquête, nous allons parler de votre quartier et de votre logement »

C1. Depuis quelle année habitez-vous dans ce logement ?

|___|___|___|___| Si vit dans ce logement depuis toujours : coder 7777
Si ne sait vraiment pas, coder 9999

C2. Le propriétaire de votre logement est :
!! Consigne : Le ménage correspond à l'ensemble des personnes résidant régulièrement dans le logement.

Vous même	1	➔ **C5.**
Un (ou plusieurs) membre du ménage appartenant à votre famille	2	➔ **C3.**
Un (ou plusieurs) membre du ménage n'appartenant pas à votre famille	3	
Un (ou plusieurs) membre de votre famille ne faisant pas partie du ménage	4	
Un ou plusieurs particuliers non membres du ménage et n'appartenant pas à votre famille	5	
Un organisme HLM (ou assimilé)	6	
Votre employeur (ou celui d'un occupant du logement)	7	
Un autre organisme privé	8	
Autre	9	

Si n'est pas personnellement propriétaire (C2 >1) :

C3. Vous personnellement, payez vous un loyer pour ce logement ?

Oui	1	➔ **C5.**
Non	2	

Si non :

C4. Quelle est votre situation ?

Vous résidez dans ce logement avec le propriétaire ou locataire principal, en tant que parent, conjoint, enfant, etc. de celui-ci	1
Vous êtes hébergé gratuitement par un (ou plusieurs) membre du ménage	2
Vous êtes hébergé par un (ou plusieurs) membre du ménage moyennant rétribution	3
Vous êtes occupant à titre gratuit	4
Vous êtes occupant sans titre (squat)	5

C5. Combien y a-t-il de pièces dans votre logement ?
!! Consigne : ne pas compter la cuisine, la salle d'eau, ni l'entrée.

|____|____| pièces

C6. Aujourd'hui, êtes-vous confronté à l'un ou plusieurs des problèmes suivants ?
!! Consigne : si non concerné aujourd'hui, coder « non pas du tout »

✎ *Carte 2*	Oui beaucoup	Oui un peu	Non pas vraiment	Non pas du tout
Logement trop petit	1	2	3	4
Logement en mauvais état (fuites dans toiture, humidité, malfaçons, etc.)	1	2	3	4
Logement trop cher	1	2	3	4

C7. Vous sentez-vous bien dans votre logement malgré ses inconvénients éventuels ?

Oui	1
Non	2

C8. Votre logement est-il localisé dans un quartier classé en Zone Urbaine Sensible (ZUS) ou, plus largement, dans un quartier dépendant de la Politique de la Ville ?
‼Consigne : ne pas expliciter les termes Zone Urbaine Sensible et politique de la ville.

Oui	1
Non	2
Ne sait pas	3

C9. Actuellement souhaitez-vous déménager ?

Oui, parce que la vie dans le quartier ne vous plait pas	1
Oui, pour une autre raison	2
Non, mais vous allez quand même devoir déménager	3
Non	4

C10. Si vous deviez déménager, regretteriez-vous :

✍ Carte 2	Oui beaucoup	Oui un peu	Non pas vraiment	Non pas du tout
Votre logement	1	2	3	4
Votre quartier	1	2	3	4
L'ambiance du quartier, les relations avec les gens	1	2	3	4
Certaines personnes du quartier en particulier	1	2	3	4
Votre ville dans son ensemble	1	2	3	4

C11. Vivez-vous depuis toujours dans ce quartier ?

Oui	1	➔ **C15.**
Non	2	

Si non :

C12. En quelle année êtes vous arrivé dans ce quartier ?
‼Consigne : la dernière fois.

|___|___|___|___|

C13. Avez-vous toujours résidé dans des quartiers du même type que celui dans lequel vous vivez aujourd'hui ?

Oui	1	➔ **C15.**
Non	2	

Si non :

C14. Avez-vous vécu :

Toujours dans des quartiers mieux (que celui où vous vivez aujourd'hui)	1
Toujours dans des quartiers moins bien	2
Dans des quartiers tantôt mieux, tantôt moins bien	3

C15. En dehors des membres de votre ménage, est-ce que des membres de votre famille habitent dans le même quartier ou la même commune que vous ?

	Oui, la plupart (plus de la moitié)	Oui, certains (moins de la moitié)	Non, aucun
Dans le même quartier	1	2	3
Hors du quartier mais dans la même commune/ le même arrondissement	1	2	3

C16. Lorsque vous, personnellement, faites les activités suivantes, à quel endroit cela se passe-t-il le plus souvent ?

!! Consigne : renseigner les activités que fait la personne enquêtée elle-même.

	Principalement dans le quartier	Principalement hors du quartier	Moitié/ moitié	Vous ne le faites pas
Faire les courses alimentaires	1	2	3	4
Utiliser les services tels que la banque, la poste	1	2	3	4
Aller chez le médecin, le pharmacien, etc.	1	2	3	4
Aller au café, au restaurant	1	2	3	4
Se promener	1	2	3	4
Rencontrer des amis	1	2	3	4
Utiliser les espaces verts, les équipements sportifs, les aires de jeux, etc.	1	2	3	4
Aller au spectacle (cinéma, concert, etc.)	1	2	3	4

C17. À propos de votre quartier, êtes vous dôaccord avec les propositions suivantes :

☞ ***Carte 3***	Tout à fait dôaccord	Plutôt dôaccord	Plutôt pas dôaccord	Pas du tout dôaccord	*NSP*
Il est loin de tout	1	2	3	4	*5*
Côest un bon endroit pour élever des enfants	1	2	3	4	*5*
Il y a beaucoup de chômage	1	2	3	4	*5*
Sa situation générale est meilleure que celle dôautres quartiers de la ville	1	2	3	4	*5*
Les transports en commun sont satisfaisants	1	2	3	4	*5*
Les écoles et établissements scolaires sont de bonne qualité	1	2	3	4	*5*
Les pouvoirs publics sôoccupent bien de ce quartier	1	2	3	4	*5*
Les commerces sont suffisamment nombreux et variés	1	2	3	4	*5*
Lôaspect de votre quartier (entretien et propreté de la voie publique, état des immeublesé) est satisfaisant	1	2	3	4	*5*
Les gens qui y vivent sôentraident facilement	1	2	3	4	*5*

C18. Dôune manière générale, quelle est la réputation de votre quartier ?

!! Consigne : Comment est-il perçu par les autres ?

Très bonne réputation	1
Bonne réputation	2
Mauvaise réputation	3
Très mauvaise réputation	4

C19. Partagez vous ce point de vue ? ☞ ***Carte 4***

Oui, tout à fait	1
Oui, plutôt	2
Non, plutôt pas	3
Non, pas du tout	4

C20. Est-ce que la réputation de votre quartier é

	Oui	Non	Sans objet
Est lôun des éléments que vous avez pris en compte pour venir habiter dans ce quartier	1	2	3<br>(habite ici depuis toujours ou nôa pas choisi dôhabiter ce quartier)
Vous a fait honte à vous personnellement	1	2	
A déjà nui à vos relations avec des collègues ou amis	1	2	
A déjà nui à votre recherche dôemploi	1	2	3

C21. Au cours des <u>deux</u> années écoulées, <u>au sein de votre quartier,</u> avez vous été :

!! Consigne : on parle bien dôévènements qui se sont produits à lôintérieur du quartier.

	Oui	Non
Personnellement victime de vol (cambriolage, vol de voiture, vol de sac à main, etc.)	1	2
Personnellement victime dôautre agression (physique, verbale, etc.)	1	2
Témoin dôagression sur dôautres personnes	1	2

C22. Au cours de lôannée écoulée, avez vous eu des contacts avec un ou plusieurs voisins et/ou dôautres personnes dans le quartier, en dehors de simples salutations (et en dehors dôamis) ?

Oui, avec des voisins	1	
Oui, avec dôautres personnes du quartier	2	
Non	3	➔ **C25**

Si oui :

C23. Avec quelle fréquence avez vous des contacts avec lôun ou lôautre de vos voisins (ou autre personne du quartier) ?

Rencontres (contacts « physiques »)		Contacts téléphoniques, courrier, mails, etc.	
|____| fois par	1- jour 2- semaine<br>3- mois 4- an	|____| fois par	1- jour 2- semaine<br>3- mois 4- an
< 1 fois par an	5	< 1 fois par an	5
Jamais	6	Jamais	6

C24. Est-ce que au moins une fois au cours de lôannée écoulée :

	Oui	Non
Vous les avez fait entrer chez vous	1	2
Vous êtes entré chez eux	1	2

C25. Que diriez-vous de vos relations avec vos voisins ? ✍ *Carte 5*

Elles sont très bonnes	1
Elles sont plutôt bonnes	2
Elles sont plutôt mauvaises	3
Elles sont très mauvaises	4
Bonnes avec certains, mauvaises avec dôautres	5
Vous nôavez aucune relation avec eux	6

C26 Vous sentez-vous très semblable, plutôt semblable, plutôt différent ou très différent des personnes qui habitent dans votre quartier ?

Très semblable	1
Plutôt semblable	2
Plutôt différent	3
Très différent	4

C27. Aujourdôhui, vous sentez vous bien dans ce quartier ? ✍ *Carte 4*

Oui, tout à fait	1
Oui, plutôt	2
Non, plutôt pas	3
Non, pas du tout	4

C28. Pouvez-vous qualifier par trois mots ou adjectifs votre quartier ?
!! Consigne : ce que votre quartier représente pour vous ?

1-

2-

3-

C29. Aujourdôhui, diriez-vous que la situation <u>générale</u> de votre quartier :
!! Consigne : Situation générale = qualité de lôenvironnement physique, social, etc.

Sôaméliore	1
Se dégrade	2
Est stable	3

C30. Vous sentez-vous en sécurité dans votre quartier ? ✍ *Carte 4*

Oui, tout à fait	1
Oui, plutôt	2
Non, plutôt pas	3
Non, pas du tout	4

« Nous allons à présent remonter dans votre passé et parler de votre parcours résidentiel »

C31. Où avez-vous passé la majeure partie de votre enfance jusquà lâge de 16 ans ?

En France (y compris Dom-Tom)	1	Département :	|___|___|
A lêtranger	2	Pays :	é é é é é é é é é .

C32. Combien de temps dans votre vie, avez-vous vécué

En France métropolitaine	|___|___| ans	Si moins dûn an, coder 00
Dont en Ile de France	|___|___| ans	Si depuis toujours, coder 97
Dont dans votre commune (ou arrondissement)	|___|___| ans	

C33. Avez-vous été sans domicile au moins une nuit dans votre vie ? (à la rue ou dans un centre dhébergement pour sans-abri, hors vacances)

Non	1	➔ **D1**.
Oui, une fois	2	
Oui, plusieurs fois	3	

Si oui :

C34. Combien de temps au total ?

|___|___| jours ou |___|___| mois ou |___|___| années

C35. La dernière fois, cêtait en quelle année ?

|___|___|___|___|

D. SANTE RESSENTIE

« A présent, nous allons aborder différentes questions concernant votre santé »

D1. Comment est votre état de santé général ? ✎ *Carte 6*

Très bon	1
Bon	2
Moyen	3
Mauvais	4
Très mauvais	5

D2. Comment est votre état de santé physique ? ✎ *Carte 6*

Très bon	1
Bon	2
Moyen	3
Mauvais	4
Très mauvais	5

D3. Comment est votre état de santé psychologique et émotionnel ? ✎ *Carte 6*

Très bon	1
Bon	2
Moyen	3
Mauvais	4
Très mauvais	5

D4. Etes-vous limité depuis au moins 6 mois à cause dûn problème de santé, dans les activités que les gens font habituellement ?

Oui, très sévèrement limité	1
Oui, sévèrement limité	2
Oui, limité	3
Non	4
(Ne sait pas)	*5*

D5. Avez-vous aujourdôhui un traitement régulier ou un suivi régulier pour raisons médicales ?

Oui	1
Non	2

D6. Avez-vous une maladie ou un problème de santé chronique (sans tenir compte des problèmes temporaires) ?

Oui	1
Non	2

D7. Avez-vous ï en dehors de vos dents de sagesseé ?

Toutes les dents intactes	1
Toutes les dents dont certaines ont été traitées ou remplacées	2
Perdu une partie des dents *(mais il vous en reste assez pour mastiquer les aliments sans nécessiter de prothèse dentaire)*	3
Perdu toutes ou presque toutes vos dents	4

D8. Actuellement, avez-vous certaines dents à soigner, en mauvais état, ou à remplacer ?

Oui	1
Non	2

D9. Dôune manière générale, habituellement, vous considérez-vous commeé

Une personne malade	1
Une personne à la santé fragile	2
Une personne en bonne santé	3
Autre : préciser ____________________________________	4

D10. Actuellement, est-ce que votre état de santé vous pose des problèmes pour travailler ?

Oui	1
Non	2

D11. Pour chacune des propositions suivantes, pouvez-vous me donner votre avis ?

✍ *Carte 3*	Tout à fait dôaccord	Plutôt dôaccord	Plutôt pas dôaccord	Pas du tout dôaccord
Vous tombez malade plus facilement que les autres	1	2	3	4
Votre corps semble très bien résister à la maladie	1	2	3	4
Quand quelque chose traîne, vous lôattrapez en général	1	2	3	4
Vous êtes facilement inquiet dès que quelque chose ne va pas	1	2	3	4
Vous avez une vie très saine	1	2	3	4
Dans lôavenir, vous vous attendez à avoir une meilleure santé que celle des autres gens que vous connaissez	1	2	3	4
Vous avez déjà été sérieusement malade	1	2	3	4
Vous pensez que votre santé sera pire dans lôavenir que maintenant	1	2	3	4
Il vous est arrivé dôêtre tellement malade que vous avez cru en mourir	1	2	3	4
Vous vous souciez davantage de votre santé que la plupart des gens	1	2	3	4
Vous évitez de penser que vous pourriez avoir un jour un cancer	1	2	3	4
Si vous aviez un cancer, vous préfèreriez le savoir le plus tard possible	1	2	3	4

E. COUVERTURE MALADIE

« Quelques questions à présent concernant votre couverture maladieé »

E1. Actuellement, avez-vous une couverture maladie ?

Oui, la Sécurité sociale de droit commun (standard)	1	
Oui, la Sécurité sociale <u>de base</u> par le biais de la CMU (Couverture maladie universelle)	2	
Oui, lœaide médicale Etat (AME)	3	
Non, aucune couverture maladie	4	➔ **E4**
(Ne sait pas)	*5*	➔ **E5**

Si oui :

E2. Etes vous pris en charge à 100% pour raison médicale ?
!! Consigne : ne concerne quœune vingtaine de maladies chroniques et/ou graves (« affections de longue durée »)

Oui	1
Non	2
(Ne sait pas)	*3*

E3. Bénéficiez-vous dœune couverture maladie complémentaire ?

Oui, par la CMU (Couverture Maladie Universelle)	1
Oui, par une mutuelle ou une assurance privée	2
Non, aucune	3
(Ne sait pas)	*4*

➔ **Aller en E5**

Si non :

E4. Depuis combien de temps nœavez-vous pas de couverture maladie ?

|___|___| mois ou |___|___| années *nœen a jamais eu, codez 99 dans la case mois*

Si moins de 3 mois ➔ ***E5***

Si plus de 3 mois ➔ ***E6***

E5. Au cours de votre vie, avez-vous déjà été au moins 3 mois sans couverture sociale maladie ?
[y compris, si vous avez vécu à lœétranger, durant cette période]

Oui	1
Non	2
(Ne sait pas)	*3*

E6. Etes-vous invalide ou handicapé ?

Oui	1	
Non	2	➔ **F1**
(Ne sait pas)	*3*	

E7. Etes-vous reconnu comme tel par la COTOREP ou la Sécurité Sociale ?

Oui	1	
Non	2	➔ **F1**
(Ne sait pas)	*3*	➔ **F1**

E8. Percevez-vous une indemnité pour cela ?

Oui	1
Non	2
(Ne sait pas)	*3*

F. RECOURS AUX SOINS

« Nous allons aborder ensuite des questions relatives aux soins médicaux »

F1. Avez-vous un médecin traitant que vous avez déclaré à la Caisse Primaire dôAssurance Maladie (Sécurité sociale) ?

Oui	1
Non mais vous savez quel médecin vous allez déclarer comme médecin traitant	2
Non et vous ne savez pas quel médecin déclarer comme médecin traitant	3
(Ne sait pas)	4

F2. Que vous lôayez déclaré ou non comme médecin traitant, avez-vous un médecin régulier qui vous connaît déjà et que vous allez consulter en priorité si vous êtes malade (médecin de famille ou médecin de quartier par exemple) ?

Oui	1	
Non	2	➔ **F4.**

Si oui :

F3. Dans quelle ville (ou arrondissement à Paris) le consultez-vous ?

Code postal (ou, par défaut, en clair) : ________________________ ➔ **Aller en F5**

Si non :

F4. Est-ce parce que ?

!! Consigne : lire chaque item

	Oui	Non
Parce que vous estimez que votre état de santé ne le nécessite pas	1	2
Parce que vous vous soignez tout seul	1	2
Parce que vous ne savez pas à qui vous adresser, ou que vous nôavez pas trouvé de médecin qui vous convienne	1	2
Pour des raisons financières (des questions dôargent)	1	2
Parce que vous nôavez pas le temps, ou vous avez dôautres soucis	1	2
Parce que les horaires de travail des médecins ne vous conviennent pas	1	2
Parce que vous nôaimez pas aller chez le médecin	1	2
Pour une autre raison :<br>précodé : vous êtes médecin ou votre conjoint est médecin ☐<br>autre : en clair ________________________________	1	2

F5. A quand remonte votre dernière visite chez un dentiste ?

Il y a un an ou moins	1	➔ **F7.**
Entre 1 et 2 ans	2	
Entre 2 et 4 ans	3	
Il y a 5 ans ou plus	4	
Vous nôavez jamais consulté de dentiste	5	

Si 2, 3, 4 ou 5 :

F6. Pour quelle raison nôavez-vous pas consulté de dentiste au cours de la dernière année ? Est-ceé

!! Consigne : Lire chaque item

	Oui	Non
Parce que vous nôen avez pas besoin	1	2
Parce que vous nôaimez pas aller chez un dentiste	1	2
Parce que vous nôavez pas le temps, ou vous avez dôautres soucis	1	2
Pour des raisons financières (des questions dôargent)	1	2
Parce que vous ne savez pas à quel dentiste vous adresser, ou que vous nôavez pas trouvé de dentiste qui vous convienne	1	2
Autre raison : en clair ________________________________	1	2

F7. Au cours des 12 dernier mois, vous est-il arrivé de renoncer, pour vous-même, à certains soins pour des raisons financières ?

!! Consigne : ne concerne pas le renoncement à des soins pour d'autres membres de la famille (enfants par exemple)

Oui	1	
Non	2	➔ **F10.**

Si oui :

F8. Pour quel(s) type(s) de soins ?

!! Consigne : Lire chaque item

	Oui	Non
Soins dentaires	1	2
Lunettes, lentilles	1	2
Séances de kinésithérapie	1	2
Consultations, visites ou soins de généralistes	1	2
Consultations, visites ou soins de spécialistes	1	2
Analyses de laboratoire, prises de sang, imagerie ou radiologie	1	2
Pharmacie, médicaments	1	2
Autres soins ou inconnus	1	2

F9. Est-ce qu'il s'agissait (au moins une fois) de soins qui vous semblaient importants ? ☞ ***Carte 4***

Oui tout à fait	1
Oui plutôt	2
Non plutôt pas	3
Non pas du tout	4

F10. Au cours des 12 derniers mois, avez-vous vu un médecin dans le cadre de la médecine du travail ?

Oui	1
Non	2
Sans objet (ne travaille pas)	3

F11. Au cours des 12 derniers mois, avez-vous eu un bilan de santé dans le cadre de la Sécurité Sociale ?

Oui	1
Non	2

F12. Au cours des 12 derniers mois, avez-vous consulté un médecin en ville ou à l'hôpital ? (non compris les hospitalisations)

!! Consigne : ne concerne que les médecins (cf. guide de l'enquêteur)

		Si oui : où était-ce ?		
		en ville	hôpital aux urgences	hôpital en consultations externes
Pour un accident	1. oui<br>2. non	1. oui<br>2. non	1. oui<br>2. non	1. oui<br>2. non
Pour un suivi, une grossesse, un renouvellement d'ordonnance	1. oui<br>2. non	1. oui<br>2. non	1. oui<br>2. non	1. oui<br>2. non
Pour une maladie ou un autre problème de santé,	1. oui<br>2. non	1. oui<br>2. non	1. oui<br>2. non	1. oui<br>2. non
En prévention (bilan, pour voir si tout va bien, vaccins, etc.)	1. oui<br>2. non	1. oui<br>2. non	1. oui<br>2. non	1. oui<br>2. non
Pour une autre raison (certificat, autreé)	1. oui<br>2. non	1. oui<br>2. non	1. oui<br>2. non	1. oui<br>2. non

F13. Au cours des 12 derniers mois, avez-vous été hospitalisé (au moins une nuit) ?

Oui	1	
Non	2	➔ **F15**

Si oui :

F14. Etait-ce au moins une fois pour ?
!! Consigne : fausse couche, IVG et avortements : coder en « opération chirurgicale »

	Oui	Non
Un accouchement	1	2
Une opération chirurgicale	1	2
Pour une autre raison	1	2

F15. Au cours des 12 derniers mois, vous est-il arrivé de consulter un médecin de votre propre initiative pour avoir un second avis médical ?
!! Consigne : Après avoir consulté un médecin une première fois

Oui	1
Non	2

F16. Toujours au cours des 12 derniers mois, avez-vous eu recours àé

	Oui	Non
Un homéopathe	1	2
Un ostéopathe	1	2
Un acuponcteur	1	2
Guérisseur traditionnel	1	2
Un autre type de médecine douce, alternative ou traditionnelle<br>Si oui : précisez_______________________	1	2

F17. Au cours des 4 dernières semaines, avez-vous consommé au moins une fois un médicament sans lôavis dôun médecin ?
!! Consigne : <u>relancer deux fois</u> :

1) on parle bien de nômporte quel médicament vendu en pharmacie, avec ou sans ordonnance
2) même pas dôaspirine, de médicaments contre la douleur ? ; pour les femmes : même pas la pilule ?

Oui	1	
Non	2	➔ **F19**

Si oui :

F18. Etait-ce au moins une fois un médicament vendu uniquement sur ordonnance ?

Oui	1
Non	2
(Ne sait pas)	*3*

F19. Au cours des 4 dernières semaines, avez-vous consommé des compléments alimentaires, des vitamines, des minéraux ?

Oui	1
Non	2
(Ne sait pas)	*3*

F20. Lôétat de santé ou la maladie de lôun de vos proches vous préoccupe-t-il particulièrement en ce moment ? ☞ ***Carte 7***

Oui, beaucoup	1
Oui, un peu	2
Non, pas vraiment	3
Non, pas du tout	4

F21. Dans votre ménage, à votre connaissance, y a-t-il des personnes qui ont actuellementé ?

	Oui	Non	Sans objet (vit seul)
Une maladie grave	1	2	3
Un handicap	1	2	3

G. HABITUDES DE VIE

« Parlons à présent de quelques-unes de vos habitudes de vieé »

G1. A quelle fréquenceé

!! Consigne : si 1 fois par semaine, cochez plusieurs fois par mois
!! Consigne : les travaux ménagers sont comptés comme « un travail » si la personne les considère comme tels
!! Consigne : la marche est considérée comme une activité physique si au moins 30 minutes dôaffilée

✎ *Carte 8*	Presque tous les jours	Plusieurs fois par semaine	Plusieurs fois par mois	Plus rarement ou jamais	Sans objet
Mangez-vous des fruits ou des légumes crus ?	1	2	3	4	
Consommez-vous de la viande ?	1	2	3	4	
Consommez-vous du poisson ?	1	2	3	4	
Dormez-vous au moins 7 heures par nuit ?	1	2	3	4	
Travaillez-vous plus de 10 heures par jour ?	1	2	3	4	5
Avez-vous une activité physique dôau moins 30 minutes dôaffilée dans le cadre de votre travail ?	1	2	3	4	5
Avez-vous une activité physique dôau moins 30 minutes dôaffilée en dehors de votre travail ?	1	2	3	4	
Prenez-vous des médicaments pour vous détendre (tranquillisants), pour dormir (somnifères) ou des anti-dépresseurs ? (hors homéopathie)	1	2	3	4	
Prenez-vous autre chose pour ces mêmes raisons : homéopathie, herbes, tisane, ou tout autre remède ?	1	2	3	4	

G2. Pratiquez-vous un sport ?

Oui, en compétition	1
Oui, régulièrement (au moins 1 fois par semaine) en amateur	2
Oui, régulièrement mais moins souvent	3
Oui, mais très occasionnellement (par exemple en vacances)	4
Non	5

G3. Vous arrive-t-il de boire du vin, de la bière ou de lôalcool ?

Oui	1	
Non	2	➔ **G8**

***Si oui :* Au cours des 6 derniers moisé .**

G4. Avez-vous ressenti le besoin de diminuer votre consommation de boissons alcoolisées ?

Oui	1
Non	2

G5. Votre entourage vous a-t-il fait des remarques au sujet de votre consommation de boissons alcoolisées ?

Oui	1
Non	2

G6. Avez-vous déjà eu lôimpression que vous buviez trop ?

Oui	1
Non	2

G7. Avez-vous eu besoin dôalcool dès le matin pour vous sentir en forme ?

Oui	1
Non	2

G8. Est-ce que vous fumez, ne serait-ce que de temps en temps ?

Oui, vous fumez quotidiennement (au moins une cigarette par jour)	1	
Oui, vous fumez occasionnellement	2	➔ **H1**
Non, vous ne fumez plus	3	➔ **G13**
Non, vous nôavez jamais fumé	4	➔ **H1**

<u>Si 1 (fume quotidiennement) :</u>

G9. Depuis combien de temps fumez-vous régulièrement ?

|___|___| ans (si moins dôun an, codez 00)

G10. Fumez-vousé . *(plusieurs réponses possibles)*

	Combien par jour en moyenne	
Des cigarettes (achetées ou tabac à rouler)	|___|___|	si variable : moyenne sur le dernier mois
Des cigares	|___|___|	si ne sait vraiment pas : coder 99
La pipe	|___|___|	

G11. Le matin, combien de temps après votre réveil fumez-vous votre première cigarette ?

Dans les 5 premières minutes	1
Entre 6 et 30 minutes	2
Entre 31 et 60 minutes	3
Après 1 heure	4
Ne sait pas	*5*

G12. Avez-vous déjà essayé dôarrêter de fumer ?

Oui	1
Non	2

➔ **Aller en H1**

<u>Si 3 (ne fume plus) :</u>

G13. Depuis combien de temps avez-vous arrêté de fumer ?

|__|__| ans (si moins dô1 an, coder 00)

H. SANTE ET MALADIES

« Nous allons à présent nous intéresser plus en détail à votre santé »

H1. Au cours de votre vie, avez-vous eu des problèmes de santé, des accidents corporels ou des maladiesé

	Oui	Non
Qui ont entraîné une interruption ou réorientation professionnelle ou de scolarité	1	2
Qui ont entraîné de graves problèmes dôargent	1	2
Qui ont entraîné une rupture avec votre conjoint ou partenaire de vie	1	2
Qui ont entraîné un autre changement important dans vos habitudes de vie	1	2

H2. Au cours de votre vie, avez-vous été victime dôaccidents du travail (signalés ou non à la Sécurité sociale) ?

Oui	1	
Non	2	➔ **H4**

<u>Si oui :</u>

H3. Combien dôaccidents du travail avez-vous eu au total ?

|___|___| accidents *(si ne sait vraiment pas, coder 99)*

H4. Pour chacune des maladies suivantes, quel est selon vous le risque que vous en soyez affecté dans les 10 ans qui viennent ? é

!! Consigne : ne pas citer NSP et relancer (en dernier recours)

✍ *Carte 9*	Très fort	Assez fort	Assez faible	Très faible ou nul	Vous en êtes actuellement atteint	*NSP*
Maladies cardiaques	1	2	3	4	5	*6*
Cancer du colon	1	2	3	4	5	*6*
Maladie de la Vache Folle	1	2	3	4	5	*6*
Sida	1	2	3	4	5	*6*
Dépression	1	2	3	4	5	*6*
Diabète	1	2	3	4	5	*6*
Maladie dôAlzheimer	1	2	3	4	5	*6*
Tuberculose	1	2	3	4	5	*6*
Cancer du sein	1	2	3	4	5	*6*

H5. Quel est votre poids ?

!! Consigne : Si femme enceinte :« quel était votre poids avant votre grossesse ?»

|___|___|___| kg (si ne sait vraiment pas, coder 999)

H6. Quelle est votre taille ?

|___|___|___| cm (si ne sait vraiment pas, coder 999)

H7. Avez-vous (vous-même ou quelquôun de votre entourage proche) déjà été victime dôune erreur médicaleé ?

Oui, tout à fait	1	
Oui, en quelque sorte	2	
Non	3	➔ **H9**

Si oui :

H8. Vous-mêmeé

	Oui	Non
En avez-vous parlé à une association de malades ?	1	2
En avez-vous parlé au médecin ou au personnel du centre de soins responsable de lôerreur ?	1	2
En avez-vous parlé à dôautres médecins ?	1	2
Avez-vous porté plainte en justice ou au Conseil de lôOrdre ?	1	2

H9. Selon vous, aujourdôhui, est-ce que certaines choses dans votre vie nuisent à votre santé physique, psychologique ou à votre moral ?

Oui	1	
Non	2	➔ **H11**

Si oui :

H10. Lesquelles ?

!! Consigne : ne pas citer, prendre les 3 premières réponses.

Ses conditions de logement	1	Sa consommation de tabac, dôalcool ou de drogues	10
Le bruit	2	Lôisolement	11
La pollution	3	Des conflits avec certains de ses proches	12
Ses conditions de travail	3	Des conflits avec dôautres personnes que ses proches (voisinsé)	13
Des mauvaises relations au travail	4	Des problèmes de santé de ses proches	14
Le stress, le rythme de vie en général	5	Son propre état de santé	15
Des problèmes économiques ou financiers	6	Dôautres problèmes familiaux	16
Des conditions de vie dans son quartier (insécurité, saleté, etc.)	7	Autre (en clair) ____________________	17
Son alimentation	8	____________________	
Le manque dôexercice physique	9	____________________	

H11. Voici une liste de problèmes de santé. Avez-vous déjà eu lõun dõentre eux ?

			**En avez-vous souffert au cours des 12 derniers mois ?**	**Ce problème a-t-il été diagnostiqué par un médecin ?**	**Avez-vous pris des médicaments ou avez-vous eu un traitement pour ce problème au cours des 12 derniers mois ?**
Asthme allergique	1. oui<br>2. non<br>3. NSP	***<u>Si oui</u>***	1. oui<br>2. non	1. oui<br>2. non	1. oui<br>2. non
Asthme non allergique	1. oui<br>2. non<br>3. NSP	***<u>Si oui</u>***	1. oui<br>2. non	1. oui<br>2. non	1. oui<br>2. non
Allergie autre quõun asthme allergique	1. oui<br>2. non<br>3. NSP	***<u>Si oui</u>***	1. oui<br>2. non	1. oui<br>2. non	1. oui<br>2. non
Diabète	1. oui<br>2. non<br>3. NSP	***<u>Si oui</u>***	1. oui<br>2. non	1. oui<br>2. non	1. oui<br>2. non
Cataracte	1. oui<br>2. non<br>3. NSP	***<u>Si oui</u>***	1. oui<br>2. non	1. oui<br>2. non	1. oui<br>2. non
Hypertension artérielle	1. oui<br>2. non<br>3. NSP	***<u>Si oui</u>***	1. oui<br>2. non	1. oui<br>2. non	1. oui<br>2. non
Attaque cardiaque, infarctus	1. oui<br>2. non<br>3. NSP	***<u>Si oui</u>***	1. oui<br>2. non	1. oui<br>2. non	1. oui<br>2. non
Attaque cérébrale, hémorragie cérébrale	1. oui<br>2. non<br>3. NSP	***<u>Si oui</u>***	1. oui<br>2. non	1. oui<br>2. non	1. oui<br>2. non
Bronchite chronique, emphysème	1. oui<br>2. non<br>3. NSP	***<u>Si oui</u>***	1. oui<br>2. non	1. oui<br>2. non	1. oui<br>2. non
Arthrite rhumatoïde, arthrose	1. oui<br>2. non<br>3. NSP	***<u>Si oui</u>***	1. oui<br>2. non	1. oui<br>2. non	1. oui<br>2. non
Ostéoporose	1. oui<br>2. non<br>3. NSP	***<u>Si oui</u>***	1. oui<br>2. non	1. oui<br>2. non	1. oui<br>2. non
Ulcère gastrique ou duodénal	1. oui<br>2. non<br>3. NSP	***<u>Si oui</u>***	1. oui<br>2. non	1. oui<br>2. non	1. oui<br>2. non
Cancer (tumeur maligne, y compris leucémie ou lymphome)	1. oui<br>2. non<br>3. NSP	***<u>Si oui</u>***	1. oui<br>2. non	1. oui<br>2. non	1. oui<br>2. non
Migraine ou fréquents maux de tête	1. oui<br>2. non<br>3. NSP	***<u>Si oui</u>***	1. oui<br>2. non	1. oui<br>2. non	1. oui<br>2. non
Anxiété chronique ou dépression	1. oui<br>2. non<br>3. NSP	***<u>Si oui</u>***	1. oui<br>2. non	1. oui<br>2. non	1. oui<br>2. non
Autre : précisez<br>______________________	1. oui<br>2. non<br>3. NSP	***<u>Si oui</u>***	1. oui<br>2. non	1. oui<br>2. non	1. oui<br>2. non
Autre : précisez<br>______________________	1. oui<br>2. non<br>3. NSP	***<u>Si oui</u>***	1. oui<br>2. non	1. oui<br>2. non	1. oui<br>2. non

H12. Pensez-vousé ?

✍ *Carte 4*	Oui tout à fait	Oui plutôt	Non plutôt pas	Non pas du tout
Que ce sont les médecins qui savent le mieux ce qui est bon pour un malade ?	1	2	3	4
Quõl faut de lõargent pour être en bonne santé ?	1	2	3	4
Quõl faut de lõargent pour se soigner ?	1	2	3	4
Que la maladie et la guérison dépendent de Dieu, du destin ou de la providence ?	1	2	3	4
Que la médecine a des réponses efficaces pour tous les problèmes de santé ?	1	2	3	4
Que les informations données par les médecins sont difficiles à comprendre ?	1	2	3	4
Que les conseils ou les recommandations de santé sont difficiles à appliquer dans la vie quotidienne ?	1	2	3	4
Quõl est possible dõavoir un cancer grave sans le savoir ?	1	2	3	4

H13. Si vous aviez besoin dõnformation sur le cancer, où iriez-vous les chercher en priorité ? *(Ne pas citer).*
!! Consigne : Noter le rang de citation spontanée dans la liste ci-dessous ; relancer ; 3 réponses max.

	1ère réponse	2ème réponse	3ème réponse
Des livres	1	1	1
Des brochures	2	2	2
Des membres de votre famille	3	3	3
Des amis ou des collègues	4	4	4
Chez mon (ou un) médecin en ville	5	5	5
A lõhôpital (ou chez un médecin à lõhôpital)	6	6	6
Sur Internet	7	7	7
Auprès dõune association	8	8	8
Des magazines	9	9	9
Des journaux	10	10	10
La radio ou la télévision	11	11	11
Des lignes téléphoniques (« SOS cancer »)	12	12	12
Autres (en clair) ____________________	13	13	13
Autres (en clair) ____________________	14	14	14
Autres (en clair) ____________________	15	15	15
Ne sait pas	*16*	*16*	*16*

H14. Des personnes de votre entourage ont-elles eu un cancer ?

Oui	1
Non	2
(Ne sait pas)	*3*

H15. Etes-vous à jour de votre vaccination contre le tétanos ?

Oui, vous en êtes certain	1
Oui, vous pensez	2
Non, vous ne croyez pas	3
Non, pas du tout	4
(Ne sait pas)	*5*

H16. Avez-vous déjà fait un test de dépistage contre le VIH/sida ?

Oui	1	
Non	2	➔ **H19**
(Ne sait pas)	*3*	➔ **H19**

Si oui :

H17. Etait-ce au moins une fois ?

	Oui	Non
A votre demande	1	2
Un examen systématique (grossesse, mariage, don de sang, etc.)	1	2
Sur les conseils d'un médecin	1	2

H18. A quand remonte votre dernier test de dépistage contre le VIH-sida ?
!! Consigne : si NSP, relancer et prendre une réponse même approximative
C'était il y a |___|___| années (si NSP, notez 99 ; si moins d'un an, notez 00)

H19. Savez-vous ce qu'est un « Hémocult » ?

Oui, tout à fait	1
Oui, à peu près	2
Non	3

H20. Avez-vous déjà eu une recherche de sang occulte dans les selles, c'est-à-dire au laboratoire ou avec une bandelette chez le médecin ?

Oui	1	
Non	2	➔ **H23**
(Ne sait pas)	*3*	➔ **H23**

Si oui :

H21. A quand remonte la dernière ?

C'était il y a |___|___| années (si NSP, coder 99, si moins d'un an, coder 00)

H22. Etait-ce ?

	Oui	Non	*NSP*
Parce que vous aviez des saignements ou d'autres troubles	1	2	*3*
Dans le cadre d'un dépistage systématique (sans que vous ayez de symptômes particuliers)	1	2	*3*
Pour une autre raison	1	2	*3*

H23. Avez-vous déjà eu une coloscopie ou une sigmoïdoscopie ? *(long tuyau qu'on enfile par en bas pour examiner l'intérieur des intestins)*

Oui	1	
Non	2	➔ **H25**
(Ne sait pas)	*3*	➔ **H25**

Si oui :

H24. A quand remonte la dernière ?
C'était il y a |___|___| années (si NSP, coder 99, si moins d'un an, coder 00)

H25. Avez-vous déjà eu un dosage du cholestérol dans le sang ?

Oui	1	
Non	2	➔ **H29**
(Ne sait pas)	*3*	➔ **H29**

Si oui :

H26. A quand remonte le dernier ?

C'était il y a |___|___| années (si NSP, coder 99, si moins d'un an, coder 00)

H27. Quel était le résultat ?

Vous aviez trop de cholestérol	1
C'était normal	2
Vous ne savez plus	3

H28. Actuellement, prenez-vous un traitement contre le cholestérol ?

Oui	1	
Non	2	
(Ne pas citer)Ne sait pas	*3*	

H29. Avez-vous déjà ressenti une douleur ou une gêne dans la poitrine ?
!! Consigne : on ne parle pas des seins

Oui	1	➔ **H31**
Non	2	

Si non :

H30. Avez-vous déjà ressenti une sensation de poids ou de lourdeur dans la poitrine ?
!! Consigne : on ne parle pas des seins

Oui	1	➔ **H31**
Non	2	➔ **H37**

Si oui à H29 ou à H30 :

H31. Apparaît-elle lorsque vous marchez vite ou que vous montez une côte ?

Oui	1	
Non	2	➔ **H37**
Non concerné	3	

H32. Apparaît-elle lorsque vous marchez d'un pas normal en terrain plat ?

Oui	1	
Non	2	
Non concerné	3	

Si au moins un Oui à H31 ou H32 ➔ **H33**
Si non ➔ **H37**

H33. Que faites-vous quand survient cette douleur ou cette gêne quand vous marchez ?

Vous vous arrêtez ou vous ralentissez	1	
Vous continuez	2	➔ **H37**
Vous prenez de la trinitrine	3	

H34. Si vous vous arrêtez, que devient la douleur ?

Elle disparaît	1	
Elle ne disparaît pas	2	➔**H 37**

H35. En combien de temps disparaît-elle ?

10 minutes ou moins	1	
Plus de 10 minutes	2	➔ **H37**

H36. Pouvez-vous me montrer où siège cette douleur ? *(plusieurs réponses possibles)*
!! Consigne : relancez une fois : « la ressentez-vous ailleurs ? ».

Haut ou milieu du sternum	1
Bas du sternum	2
Paroi thoracique avant gauche (sous le cœur)	3
Bras gauche	4
Mâchoires	5
Epaules	6
Autres localisations : (Précisez)<br>……………………………………………… .<br>……………………………………………… ..	7

H37. Ressentez-vous une douleur ou une gêne dans une jambe (ou dans les jambes) quand vous marchez ?

Oui	1	
Non	2	→ **I1**
Non concerné	3	→ **I1**

Si oui :

H38. Cette douleur commence-t-elle parfois à se manifester quand vous êtes assis ou debout immobile ?

Oui	1	→ **I1**
Non	2	

Si non :

H39. Ressentez-vous cette douleur quand vous montez une côte ou quand marchez vite ?

Oui	1	
Non	2	→ **I1**
Non concerné	3	→ **I1**

Si oui :

H40. La ressentez-vous quand vous marchez d'un pas normal sur terrain plat ?

Oui	1
Non	2

H41. Que devient la douleur si vous vous arrêtez ?

Elle persiste habituellement plus de 10 minutes	1	→ **I1**
Elle disparaît habituellement en 10 minutes ou moins	2	

Si disparaît en moins de 10 minutes :

H42. Où ressentez-vous cette douleur ou cette gêne ?
(plusieurs réponses possibles)

Mollets	1
Fesses	2
Cuisses	3
Articulations (genoux, hanches, chevilles, etc.)	4

I. SANTE PSYCHOLOGIQUE

« Nous abordons maintenant quelques questions sur la santé psychologique… »

I1. Au cours des deux dernières semaines…
!! Consigne : lire chaque item

	Oui	Non
Vous êtes-vous senti particulièrement triste, cafardeux, déprimé, la plupart du temps au cours de la journée, et ce, presque tous les jours ?	1	2
Aviez-vous presque tout le temps le sentiment de n'avoir plus goût à rien, d'avoir perdu l'intérêt ou le plaisir pour les choses qui vous plaisent habituellement ?	1	2
Vous sentiez-vous presque tout le temps fatigué, sans énergie ?	1	2

Si au moins 2 "oui": poser la question suivante

Sinon : → I5

I2. Au cours de ces deux dernières semaines, lorsque vous vous sentiez déprimé(e) / sans intérêt pour la plupart des choses / fatigué(e) :é

!! Consigne : lire chaque item

	Oui	Non
Votre appétit a-t-il notablement changé <u>ou</u> avez-vous pris ou perdu du poids sans en avoir lõintention ?<br>*!! Consigne : Coder **OUI,** si **OUI** à lõun ou lõautre*	1	2
Aviez-vous des problèmes de sommeil (endormissement, réveils nocturnes ou précoces, hypersomnie) presque toutes les nuits ?	1	2
Parliez-vous ou vous déplaciez-vous plus lentement que dõhabitude, ou au contraire vous sentiez-vous agité(e) et aviez-vous du mal à rester en place ?	1	2
Manquiez-vous de confiance en vous-même, ou vous sentiez-vous sans valeur, voire inférieur(e) aux autres ?	1	2
Vous êtes-vous fait des reproches, ou vous êtes-vous senti(e) coupable ?	1	2
Avez-vous eu du mal à réfléchir ou à vous concentrer, ou aviez-vous du mal à prendre des décisions ?	1	2
Avez-vous eu à plusieurs reprises des idées noires comme penser quõil vaudrait mieux que vous soyez mort(e), ou avez-vous pensé à vous faire du mal ?	1	2

<u>Si au moins 4 "oui" en I2 :</u> poser la question suivante

<u>Sinon :</u> ➔ Aller à la partie J

I3. En avez-vous parlé à un professionnel de la santé, ou êtes-vous déjà suivi(e) pour cela ?

Oui	1	
Non	2	**➔ J1**

<u>Si oui :</u>

I4. Qui est ce professionnel ? *(plusieurs réponses possibles)*

Médecin généraliste	1
Psychiatre	2
Psychologue ou psychanalyste	3
Homéopathe	4
Autre médecin	5
Pharmacien	6
Médecin traditionnel (médecine chinoise, guérisseur, etc.)	7
Autre	8

➔ Aller à la partie suivante J

I5. Au cours des deux dernières semainesé

!! Consigne : lire chaque item

	Oui	Non
Avez-vous perdu lõappétit ?	1	2
Avez-vous des problèmes de sommeil (insomnie, réveils nocturnes ou précoces, hypersomnie) presque toutes les nuits ?	1	2
Vous sentez-vous presque tout le temps fatigué, sans énergie ?	1	2
Vous sentez-vous presque tout le temps bon à rien ou coupable ?	1	2
Avez-vous du mal à vous concentrer et à prendre des décisions ?	1	2

J. SANTE DES FEMMES

Si homme : ***➔ passer directement à la partie K (K1)***
Si femme :

« Nous allons maintenant aborder un chapitre concernant les femmes »

J1. Etes-vous actuellement enceinte ?

Oui	1	
Non	2	**➔ J3**

Si est enceinte :

J2. Avant votre grossesse, étiez-vous régulièrement suivie pour les questions de gynécologie ?

Oui	1	**➔ J4**
Non	2	**➔ J6**

Si nœst pas enceinte :

J3. Etes-vous régulièrement suivie pour les questions de gynécologie ?

Oui	1	**➔ J4**
Non	2	**➔ J6**

Si est/était suivie :

J4. Par qui ?

Un généraliste en ville	1
Un gynécologue en ville	2
Un médecin à lœhôpital	3
Autre (dispensaire, centre de soins, etc.)	4
(Ne sait pas)	*5*

J5. Dans quelle ville (ou arrondissement à Paris) le consultez-vous ?
Code postal (ou, par défaut, en clair) : ________________________

Si nœst / nœtait pas suivie :

J6. Pour quelle raison ? Est-ce (était-ce)é

	Oui	Non
Parce que vous ne savez (saviez) pas à qui vous adresser, ou vous nœavez (nœaviez) pas trouvé de médecin qui vous convienne	1	2
Parce que vous nœn avez (nœaviez) pas besoin	1	2
Parce que vous nœavez (nœaviez) pas le temps, ou vous avez (aviez) dœautres soucis	1	2
Pour des raisons financières (des questions dœargent)	1	2
Parce que vous nœaimez (nœaimiez) pas ou ne voulez (vouliez) pas dœexamens gynécologiques	1	2
Autre : en clair ______________________________________	1	2

J7. Avez-vous déjà eu une mammographie ? *(cœst-à-dire une radiographie des seins)*

Oui	1	
Non	2	**➔ J12**
Ne sait pas, nœst pas sûre	*3*	**➔ J13**

Si oui :

J8. A quand remonte la dernière ?

Cœtait il y a |___|___| années (si NSP, coder 99, si moins dœun an, coder 00)

J9. Cette dernière fois, comment s’est passé l’examen ?

Très bien	1
Assez bien	2
Assez mal	3
Très mal	4

J10. Cette dernière fois, était-ce :

A votre demande	1
Sur les conseils d’un médecin	2
En recevant une lettre d’invitation pour le dépistage	3
Autre	4
(Ne sait pas)	*5*

J11. Toujours cette dernière fois, aviez-vous des symptômes qui vous inquiétaient ou inquiétaient votre médecin ?

Oui	1
Non	2
(Ne sait pas)	*3*

<u>Si la dernière mammographie remonte à plus de 3 ans et âge supérieur ou égal à 40 ans</u> ➔ J12

<u>Si remonte à 3 ans ou moins, ou si âge strictement inférieur à 40 ans</u> ➔ J13

J12. Pour quelles raisons
N’avez-vous jamais eu de mammographie ?
ou **N’avez-vous pas fait de mammographie depuis cette date ?**

	Oui	Non
Parce que vous ne savez pas où le faire	1	2
Parce que vous n’aimez pas cet examen	1	2
Parce que personne ne vous l’a proposé	1	2
Pour des raisons financières (des questions d’argent)	1	2
Parce que vous n’avez pas le temps, vous avez d’autres soucis	1	2
Parce que vous redoutez les résultats, vous préférez ne pas connaître les résultats	1	2
Parce que vous n’en avez pas besoin, ou n’êtes pas concernée	1	2

J13. Un médecin vous a-t-il déjà montré comment vous palper vous-même les seins ?

Oui	1
Non	2
Ne sait pas	*3*
Vous êtes médecin	*4*

J14. Vous palpez-vous les seins vous-même ?

Oui régulièrement	1
Oui parfois	2
Non rarement	3
Non jamais	4

J15. Avez-vous déjà eu un frottis du col de l’utérus ou un frottis vaginal ? (petit prélèvement en frottant au fond du vagin, pendant un examen gynécologique)

Oui	1	
Non	2	➔ **J17**
Ne sait pas	*3*	➔ **K1**

<u>Si oui :</u>

J16. A quand remonte le dernier ?

|___|___| ans (si NSP, coder 99, si moins d’un an, coder 00)

<u>Si 3 ans ou plus ou NSP,</u> ➔ Aller en J17

<u>Si moins de 3 ans,</u> ➔ Aller en K1

J17. Pour quelles raisons
Nôavez-vous jamais eu de frottis ? ***ou*** **Nôavez-vous pas eu de frottis depuis cette date ?**

	Oui	Non
Parce que vous ne savez pas où le faire	1	2
Parce que vous nôaimez pas cet examen	1	2
Parce que personne ne vous lôa proposé	1	2
Pour des raisons financières (des questions dôargent)	1	2
Parce que vous nôavez pas le temps, vous avez dôautres soucis	1	2
Parce que vous redoutez les résultats, vous préférez ne pas savoir	1	2
Parce que vous nôen avez pas besoin, tout va bien	1	2
Ne pas citer : Hystérectomie, nôa plus dôutérus	1	

K. INFORMATION EN MATIERE DE SANTE

« Nous allons à présent parler de vos habitudes et de vos besoins en matière dôinformation sur la santé »

K1. Y a-t-il parmi vos connaissances des personnes qui travaillent dans le milieu médical et à qui vous pourriez demander conseil en cas de besoin ?

Oui, lôun de vos proches	1
Oui, quelquôun de moins proche	2
Non, personne	3

K2. Dôune manière générale, êtes-vous intéressé par les informations qui portent sur la santé et la médecine ?

Très intéressé	1
Plutôt intéressé	2
Plutôt pas intéressé	3
Pas intéressé du tout	4

K3. Lorsque vous vous posez une question sur un sujet de santé, auprès de qui ou comment obtenez-vous des renseignements ?

	La plupart du temps	Assez Souvent	Rarement	Jamais
Auprès dôun médecin	1	2	3	4
Auprès dôun pharmacien, ou dôun autre professionnel de santé	1	2	3	4
Auprès de votre famille ou dôamis	1	2	3	4
Par la télévision, la radio, ou la presse générale	1	2	3	4
En lisant une revue ou un magazine spécialisé sur la santé	1	2	3	4
En cherchant sur Internet	1	2	3	4

K4. Au cours des 3 dernières années, en dehors des consultations médicales, avez-vous cherché des informations ou des conseils suré

	Non	Oui		
		uniquement sur Internet	jamais sur Internet	à la fois sur Internet et par un autre moyen
Lôactualité médicale, les nouveautés en matière de santé	1	2	3	4
Les centres de soins, hôpitaux ou médecins	1	2	3	4
Une maladie ou un symptôme particulier	1	2	3	4
Un médicament ou un traitement médical	1	2	3	4
Des médecines ou remèdes alternatifs, traditionnels, ou la médecine douce	1	2	3	4
La diététique, les régimes	1	2	3	4
La dépression, lôanxiété, le stress, la santé mentaleé	1	2	3	4
Des démarches liées au système de protection sociale (Sécurité sociale, déclaration dôaccidents du travail, reconnaissance dôun handicap, etc.)	1	2	3	4

Si « non » (1) ou « oui, jamais sur Internet » (3) partout ➔ ***aller en*** **K5**
Si au moins une fois sur Internet (2ou 4)➔ ***aller en*** **K6**

K5. Vous êtes-vous néanmoins déjà renseigné sur dôautres sujets concernant la santé sur Internet ?

Oui	1	
Non	2	➔ **K7**
Ne sait pas	*3*	➔ **K7**

Si oui :

K6. Quand vous avez cherché des informations concernant la santé sur Internet, était-ce au moins une fois pour avoir des informationsé

	Oui	Non
A la place dôaller chez le médecin	1	2
Avant dôaller consulter un médecin	1	2
Après une consultation médicale	1	2
Sans lien avec une consultation médicale	1	2

Si non :

K7. Pour quelle raison nôavez-vous jamais cherché ce type dôinformation sur Internet ?
!!Consigne : ne rien suggérer, (plusieurs réponses possibles)

Pas dôaccès à Internet	1
Pas intéressé en ce moment	2
Ne sait pas vraiment comment effectuer la recherche sur Internet (ou côest trop compliqué)	3
Nôa pas confiance en lôinformation diffusée sur Internet	4
Pense quôil vaut mieux aller voir un médecin pour toutes ces questions	5
Sources habituelles dôinformation sur la santé suffisantes	6
Nôy a jamais pensé	7
Autre : précisez ________________________________	8
________________________________	

L. OPINIONS SUR SA VIE PERSONNELLE

« La prochaine partie porte sur les opinions que vous pouvez avoir sur vous-même, dans différents domaines »

L1. Voici différentes propositions concernant votre sentiment général sur vous-même.
Pour chacune, pouvez vous me dire si vous êtes tout à fait dôaccord, plutôt dôaccord, plutôt pas dôaccord, pas dôaccord du tout ?

✎ *Carte 3*	Tout à fait dôaccord	Plutôt dôaccord	Plutôt pas dôaccord	Pas du tout dôaccord
Dans lôensemble, vous êtes satisfait de vous	1	2	3	4
Parfois vous pensez que vous ne valez rien	1	2	3	4
Vous pensez que vous avez un certain nombre de bonnes qualités	1	2	3	4
Vous êtes capable de faire les choses aussi bien que la plupart des gens	1	2	3	4
Vous pensez qu'il n'y a pas grand-chose en vous dont vous pouvez être fier	1	2	3	4
Parfois, vous vous sentez réellement inutile	1	2	3	4
Vous pensez que vous êtes quelqu'un de valable, au moins autant que les autres	1	2	3	4
Vous aimeriez pouvoir avoir plus de respect pour vous-même	1	2	3	4
Vous avez tendance à penser que vous êtes un(e) raté(e)	1	2	3	4
Vous avez une opinion positive de vous-même	1	2	3	4

L2. Pour vous, dans votre vie dôaujourdôhui, quelle importance accordez-vous à ces différentes choses (même si cela ne correspond pas à votre situation actuelle) ?

	Très important	Plutôt important	Plutôt pas important	Pas du tout important
Avoir des enfants	1	2	3	4
Vivre avec un partenaire avec lequel on sôentend bien	1	2	3	4
Avoir un travail intéressant	1	2	3	4
Voir des amis régulièrement	1	2	3	4
Avoir des loisirs ou des activités culturelles (lecture, cinémaé)	1	2	3	4
Prendre des vacances	1	2	3	4
Etre utile aux autres	1	2	3	4
Avoir du temps pour soi (pour sôoccuper de soi)	1	2	3	4
Se sentir reconnu dans la société	1	2	3	4
Avoir une bonne santé	1	2	3	4
Avoir une bonne alimentation (suffisante et équilibrée)	1	2	3	4

L3. Concernant ces différents points, dans votre vie actuelle, êtes-vous très satisfait, plutôt satisfait, plutôt pas satisfait ou pas satisfait du tout ?

☞ *Carte 14*	Très satisfait	Plutôt satisfait	Plutôt pas satisfait	Pas satisfait du tout	Sans objet
Votre vie amoureuse	1	2	3	4	
Votre situation de famille	1	2	3	4	
Votre situation professionnelle (par rapport à lôemploi)	1	2	3	4	5
Vos relations amicales	1	2	3	4	
Vos loisirs et activités culturelles	1	2	3	4	
Vos vacances	1	2	3	4	
Votre santé	1	2	3	4	
Votre alimentation	1	2	3	4	
Le temps que vous avez pour vous occuper de vous	1	2	3	4	

M- ENFANCE ET JEUNESSE

« Nous allons à présent revenir sur votre enfance et votre jeunesse »

M1. Entre votre naissance et lôâge de 16 ans inclus, par qui avez-vous été élevé (pour une durée dôau moins un an) ?
!! Plusieurs réponses possibles, si la situation a changé

Vos deux parents biologiques en couple	1
Votre mère biologique seule	2
Votre père biologique seul	3
Votre mère biologique et un(e) conjoint(e)	4
Votre père biologique et un(e) conjoint(e)	5
Un autre couple (à qui vous avez été confié ou qui vous a adopté) : (Précisez)	
Un couple de votre famille (grands parents, oncle et tante, é)	6
Un couple ne faisant pas partie de votre famille et qui vous a adopté	7
Un couple ne faisant pas partie de votre famille à qui vous avez été confié	8
Une autre femme seule : (Précisez)	
Une femme de votre famille (grand-mère, tanteé)	9
Une femme ne faisant pas partie de votre famille et qui vous a adopté	10
Une femme ne faisant pas partie de votre famille et à qui vous avez été confié	11

Un autre homme seul : (Précisez)	
Un homme de votre famille (grand-père, oncleé)	12
Un homme ne faisant pas partie de votre famille et qui vous a adopté	13
Un homme ne faisant pas partie de votre famille et à qui vous avez été confié	14
Un établissement collectif (orphelinat, foyer, pensionnaté)	15
Autre : précisez ____________________	16

Si plusieurs réponses :

M2. A lôâge de 16 ans, par qui étiez vous élevé ?

Vos deux parents biologiques en couple	1
Votre mère biologique seule	2
Votre père biologique seul	3
Votre mère biologique et un(e) conjoint(e)	4
Votre père biologique et un(e) conjoint(e)	5
Un autre couple (à qui vous avez été confié ou qui vous a adopté)	6
Une autre femme seule	7
Un autre homme seul	8
Un établissement collectif (pensionnat, orphelinat, foyer)	9
Autre : précisez ____________________	10

A tous

M3. Votre père est-il encore en vie ?

Oui	1
Non, il est décédé	2
Ne sait rien sur son père (père inconnu)	3
Ne sait pas, sans nouvelle, perdu de vue	4

➔ **Quel âge aviez vous lors de son décès** |__|__| ans
(si avant la naissance de lôenquêté, coder 99)

M4. Votre mère est-elle encore en vie ?

Oui	1
Non, elle est décédée	2
Ne sait rien sur sa mère (mère inconnue)	3
Ne sait pas, sans nouvelle, perdue de vue	4

➔ **Quel âge aviez vous lors de son décès** |__|__| ans
(si avant la naissance de lôenquêté, coder 99)

M5. Vos parents ont-ils divorcé ou se sont-ils séparés ?

Oui	1
Non	2
Sans objet (nôont jamais vécu ensemble)	3
Ne sait pas	4

➔ **Quel âge aviez vous (la 1ère fois) ?** |__|__| ans
(si avant sa naissance, coder 99)

M6. Avant lôâge de 18 ans que diriez vous des relations avec : ✍ *Carte 10*

	Votre père	Votre mère
Très bonnes	1	1
Plutôt bonnes	2	2
Plutôt mauvaises	3	3
Très mauvaises	4	4
Aucune relation	5	5
A certaines périodes bonnes, à dôautres mauvaises	6	6
Décédé, inconnu, disparu ou pas concerné	7	7

M7. Avant lõâge de 18 ans, aviez vous le sentiment de compter pour vos parents ? *Carte 11*

	Pour votre père	Pour votre mère
Oui beaucoup	1	1
Oui un peu	2	2
Non pas beaucoup	3	3
Non pas du tout	4	4
Décédé, inconnu, disparu ou pas concerné	5	5

M8. Votre père travaillait-il lorsque vous aviez 16 ans ?

Décédé, inconnu, disparu	1	
Oui	2	
Non, il ne travaillait plus à ce moment là pour cause de chômage	3	
Non, il ne travaillait plus à ce moment là pour une autre raison	4	
Non, il nõa jamais travaillé	5	➔ **M10.**

M9. Quelle était la profession (ou la dernière profession) de votre père lorsque vous aviez 16 ans (ou à son décès si décédé avant vos 16 ans) ?

Père inconnu		1
Ne sait pas		2
En clair :	é é<br>é é	3
Puis recoder avec la grille PCS INSEE :	|__________| *Carte 1*	

M10. Votre mère travaillait-elle lorsque vous aviez 16 ans ?

Décédée, inconnue, disparue	1	
Oui	2	
Non, elle ne travaillait plus à ce moment là pour cause de chômage	3	
Non, elle ne travaillait plus à ce moment là pour une autre raison	4	
Non, elle nõa jamais travaillé	5	➔ **M12.**

M11. Quelle était la profession (ou la dernière profession) de votre mère lorsque vous aviez 16 ans (ou à son décès si décédée avant vos 16 ans) ?

Mère inconnue		1
Ne sait pas		2
En clair :	é é<br>é é	3
Puis recoder avec la grille PCS INSEE :	|__________| *Carte 1*	

M12. Quelle est la nationalité de votre père *(ou quõelle était sa nationalité si décédé)* ?

!! Consigne : Plusieurs réponses possibles

Française	1
Autre : précisez ________________________________	2
Autre : précisez ________________________________	3
Autre : précisez ________________________________	4
Ne sait pas ou sans objet	*5*

M13. Quelle est la nationalité de votre mère *(ou quõelle était sa nationalité si décédée)* ?

!! Consigne : Plusieurs réponses possibles

Française	1
Autre : précisez ________________________________	2
Autre : précisez ________________________________	3
Autre : précisez ________________________________	4
Ne sait pas ou sans objet	*5*

M14. Nous allons aborder certains types dœvénements difficiles. Pouvez-vous nous dire pour chacun dœux si, avant lâge de 18 ans, vous les avez vécus et, si oui, à quel âge (la première fois)

	Non	Oui		A quelle période de votre vie ? *(plusieurs réponses possibles)*
		Vous en êtes encore marqué	*Vous les avez surmontés*	1. entre 0-6 ans<br>2. entre 7-12 ans<br>3. entre 13-18 ans
Vous avez eu de graves problèmes de santé	1	2	3	1 2 3
Vos parents ont eu de graves problèmes de santé	1	2	3	1 2 3
Votre famille a eu de graves problèmes de logement	1	2	3	1 2 3
Votre famille a eu de gros problèmes dœargent	1	2	3	1 2 3
Vous avez vécu dans une zone de guerre ou de conflit	1	2	3	1 2 3
Vous avez été suivi(e) par un éducateur	1	2	3	1 2 3
Vous avez fait une ou des fugues	1	2	3	1 2 3
Vous avez eu à faire avec la police	1	2	3	1 2 3
Vous avez été suivi(e) par un juge des enfants	1	2	3	1 2 3
Vous avez été en prison	1	2	3	1 2 3
Vous avez fait des tentatives de suicide	1	2	3	1 2 3
Lœun de vos parents est décédé	1	2	3	1 2 3
Lœun de vos frères ou sî urs est décédé	1	2	3	1 2 3
Lœun de vos parents a connu un chômage prolongé	1	2	3	1 2 3
Vos parents avaient de graves disputes	1	2	3	1 2 3
Vos parents ont divorcé (ou se sont séparés)	1	2	3	1 2 3
Vos parents (ou lœun dœux) était alcoolique	1	2	3	1 2 3
Votre père ou votre mère est allé en prison	1	2	3	1 2 3
Lœun de vos frères ou sî urs est allé en prison	1	2	3	1 2 3
Lœun de vos parents a fait des tentatives de suicide	1	2	3	1 2 3
Vous avez subi de mauvais traitements	1	2	3	1 2 3
Votre mère a subi de mauvais traitements	1	2	3	1 2 3
Vous avez été victime dœabus sexuels	1	2	3	1 2 3
Autres difficultés : *(précisez)*<br>é é é é é é é é é é é é é é é é é é<br>é é é é é é é é é é é é é é é é é é .<br>é é é é é é é é é é é é é é é é é é .	1	2	3	1 2 3

M15. Avant lâge de 18 ans, avez-vous été placé (par la DDASS, lœASE, la PJJ) dans une famille dœaccueil ou une institution ou un foyer, même provisoirement ?

Oui	1	
Non	2	➔ **M19**

Si oui :

M16. A quel âge était-ce la première fois ? |___|___| ans

M17. Au total, pendant combien de temps avez-vous vécu ? :

dans une famille dœaccueil	|___|___|	années	*(si moins dœ1 an, coder 00)*
dans une institution ou un foyer	|___|___|	années	*(si moins dœ1 an, coder 00)*

M18. La décision d'un placement en famille d'accueil et/ou en institution a été prise :

Parce que vous avez perdu vos parents	1
Suite à des faits reprochés à vos parents	2
Autre	3

M19. Avez vous eu des frères et sœurs (même s'ils sont à présent décédés) ?

Oui	1	
Non	2	➔ **M21.**

Si oui :

M20. Combien en avez vous eu et combien ont été élevés avec vous ?

|___|___| frères et sœurs dont |___|___| élevés avec moi.

« Pour conclure cette partie… »

M21. Diriez-vous que votre enfance a été heureuse ? ✎ *Carte 4*

Oui, tout à fait	1
Oui, plutôt	2
Non, plutôt pas	3
Non, pas du tout	4

M22. Diriez-vous que votre adolescence a été heureuse ? ✎ *Carte 4*

Oui, tout à fait	1
Oui, plutôt	2
Non, plutôt pas	3
Non, pas du tout	4

M23. En ce qui concerne votre vie adulte, nous allons aborder certains types d'événements difficiles. Pouvez-vous nous dire pour chacun d'eux si vous les avez déjà vécu après l'âge de 18 ans ?

	Oui	Non
Vous avez été condamné en justice	1	2
Vous avez été en prison	1	2
Vous avez été victime de violences physiques ou sexuelles	1	2
Vous avez été victime de violences psychologiques ou morales	1	2
Vous avez été régulièrement privé de liberté et d'autonomie par des membres de votre famille	1	2
Vous avez eu des problèmes avec l'alcool ou la drogue	1	2
Vous avez été hospitalisé en hôpital psychiatrique	1	2
Vous avez fait une tentative de suicide	1	2

N- VIE FAMILIALE ACTUELLE

« Nous allons à présent parler de vos relations <u>actuelles</u> avec votre famille »

<u>Si le père est encore en vie :</u>

N1. Avec quelle fréquence avez vous des contacts (y compris par téléphone) avec votre père ?

**Rencontres (contacts « physiques »)**		**Contacts téléphoniques, courrier, mails, etc.**	
|___|___| fois par	1- jour 2- semaine<br>3- mois 4- an	|___|___| fois par	1- jour 2- semaine<br>3- mois 4- an
Moins d'une fois par an	5	Moins d'une fois par an	5
Jamais	6	Jamais	6
Sans objet : vit avec lui	7	Sans objet : vit avec lui	7

<u>Si la mère est encore en vie :</u>

N2. Avec quelle fréquence avez vous des contacts (y compris par téléphone) avec votre mère ?

**Rencontres (contacts « physiques »)**		**Contacts téléphoniques, courrier, mails, etc.**	
|___|___| fois par	1- jour 2- semaine<br>3- mois 4- an	|___|___| fois par	1- jour 2- semaine<br>3- mois 4- an
Moins d'une fois par an	5	Moins d'une fois par an	5
Jamais	6	Jamais	6
Sans objet : vit avec elle	7	Sans objet : vit avec elle	7

<u>Si le père et la mère sont décédés</u> : ➔ N6.

N3. Aimeriez vous avoir des contacts avec votre père / votre mère :

	Père	Mère
Plus souvent	1	1
C'est juste ce qu'il faut	2	2
Moins souvent	3	3
Ne sait pas	4	4
(Décédé, inconnu, disparu ou pas concerné)	*5*	*5*

N4. Aujourd'hui que diriez vous des relations avec lui / elle : ✍ ***Carte 12***

	Votre père	Votre mère
Très bonnes	1	1
Plutôt bonnes	2	2
Plutôt mauvaises	3	3
Très mauvaises	4	4
Aucune relation	5	5
Décédé, inconnu, disparu ou pas concerné	6	6

N5. Aujourd'hui, avez-vous le sentiment de compter pour votre père / mère ? ✍ ***Carte 11***

	Votre père	Votre mère
Oui beaucoup	1	1
Oui un peu	2	2
Non pas beaucoup	3	3
Non pas du tout	4	4
Sans objet	5	5

<u>Si a eu des frères et sœurs :</u>

<u>Sinon ➔ O1</u>

N6. Aujourd'hui, parmi vos frères et sœurs, y en a-t-il avec qui vous avez :

	Oui	Non
De bonnes relations	1	2
De mauvaises relations	1	2
Aucune relation (*pour des raisons autres que décès, etc.*)	1	2

N7. Avec quelle fréquence avez vous des contacts (y compris par téléphone) avec l'un ou l'autre de vos frères et sœurs ?

**Rencontres (contacts « physiques »)**		**Contacts téléphoniques, courrier, mails, etc.**	
|___|___| fois par	1- jour 2- semaine<br>3- mois 4- an	|___|___| fois par	1- jour 2- semaine<br>3- mois 4- an
Moins d'une fois par an	5	Moins d'une fois par an	5
Jamais	6	Jamais	6
Vivent ensemble	7	Vivent ensemble	7

O. FORMATION

« Nous allons à présent parler de votre formation scolaireé «

O1. Dans la vie courante, lisez-vous le Françaisé

Avec beaucoup de difficultés	1
Avec quelques difficultés	2
Sans difficultés	3

O2. Toujours dans la vie courante, écrivez-vous en Françaisé

Avec beaucoup de difficultés	1
Avec quelques difficultés	2
Sans difficultés	3

O3. Jusquà quel niveau dőtudes avez-vous été ?

Jamais été à lőcole	1	➔ **P1.**
Enseignement primaire	2	
Enseignement secondaire 1er cycle (de la 6ème à la 3ème)	3	
Enseignement secondaire (deuxième cycle)	4	
Enseignement supérieur	5	

<u>Si a été à lőcole :</u>

O4. Quel est le diplôme le plus élevé que vous avez obtenu ?

Aucun diplôme	1
CEP (certificat dőtudes primaires)	2
BEPC, BE (brevet élémentaire), BEPS	3
CAP, BEP ou autre diplôme de ce niveau	4
Baccalauréat professionnel	5
Baccalauréat technique ou technologique	6
Baccalauréat général	7
Niveau Bac+2 (DEUG, BTS, IUT)	8
Bac + 3 et au-delà	9

O5.Votre famille attachait-elle de lőmportance à la réussite scolaire ?

Sans objet (sans famille)	1
Oui, beaucoup	2
Oui, un peu	3
Non, pas vraiment	4
Non, pas du tout	5

O6. Si vous deviez faire le bilan de votre scolarité, diriez-vous que vous avez aimé vos études ? ✎ *Carte 4*

Oui, tout à fait	1
Oui, plutôt	2
Non, plutôt pas	3
Non pas du tout	4

P- SITUATION ET EXPERIENCE PROFESSIONNELLES

« Nous allons maintenant poursuivre avec votre activité professionnelle »

P1. Quelle est votre situation principale actuelle ?

!! Consigne : Occupent un emploi toutes les personnes suivantes :

- *les personnes qui aident un membre de leur famille dans leur travail, même sans être rémunérées,*
- *les personnes en congé annuel de maladie, de maternité, en congé individuel de formation, de conversion, en dispenses d'activité, etc.*
- *les élèves fonctionnaires, les intérimaires, les intermittents du spectacle*

!! Consigne : si étudiant et salarié, coder étudiant

Vous occupez un emploi	1	➔ **P57.**
Vous êtes apprenti sous contrat ou en stage rémunéré	2	➔ **P2.**
Vous êtes étudiant élève, en formation ou en stage non rémunéré	3	➔ **P2.**
Vous êtes chômeur (inscrit ou non à l'ANPE)	4	➔ **P18.**
Vous êtes retraité ou retiré des affaires ou en préretraite	5	➔ **P7.**
Vous êtes femme ou homme au foyer	6	➔ **P39.**
Vous êtes en congé parental à temps plein	7	➔ **P57.**
Autre situation d'inactivité (personne handicapée…)	8	➔ **P39.**
Autre situation	9	➔ **(à évaluer)**

Si étudiant, élève, en formation ou stage, apprenti sous contrat

P2. Avez-vous repris des études ou une formation après interruption, ou êtes-vous en cours d'études initiales ?

Etudes ou formation après interruption	1
Etudes ou formation initiales	2

P3. Aujourd'hui, avez-vous une activité professionnelle, de quelque type que ce soit (salarié, indépendant, petits boulots, etc.) …

De façon régulière, à longueur d'année	1	
De façon saisonnière	2	➔ **P5.**
De façon occasionnelle ou exceptionnelle	3	➔ **P5.**
Jamais	4	➔ **P5.**

Si a une activité professionnelle régulière :

P4. Combien d'heures par mois consacrez-vous à cette activité professionnelle ?

[___|____|___] Si plus de 40 heures par mois : ➔**Aller en P57**

P5. Actuellement, recherchez-vous un emploi ?

Oui	1	
Non	2	➔ **Q1**

Si oui :

P6. Pensez-vous que vous aurez prochainement un emploi ? ☞ *Carte 4*

Oui, tout à fait	1
Oui, plutôt	2
Non, plutôt pas	3
Non, pas du tout	4
(Ne sait vraiment pas)	*5*

➔ **Aller en partie Q**

Si retraité ou pré-retraité

P7. Depuis quelle date êtes-vous en retraite ou pré-retraite ?

Mois |__|__| et Année |__|__|__|__|

P8. Auparavant, quelle profession exerciez-vous (celle qui a duré le plus longtemps) ?

En clairé .é é é é é é é é é é é é ..
é .é é

Puis recoder : ✐ *Carte 1* |__|__|

P9. A lœpoque, étiez-vousé

Salarié de lŒtat	1
Salarié dœune collectivité locale, des HLM ou des hôpitaux publics	2
Salarié dœune entreprise, dœun artisan, dœune association	3
Salarié chez un particulier	4
Vous aidiez un membre de votre famille dans son travail sans être rémunéré	5
Chef dœntreprise salarié, PDG, gérant minoritaire, associé	6
Indépendant ou à votre compte	7
Autre	8

P10. Dans quel secteur dœactivité était-ce ? ✐*Aide enquêteur : Carte 13*

Agriculture, sylviculture, pêche	1
Industries agricoles et alimentaires	2
Industrie des biens de consommation	3
Industrie automobile	4
Industries des biens d'équipement	5
Industries des biens intermédiaires	6
Energie	7
Construction	8
Commerce	9
Transports	10
Activités financières	11
Activités immobilières	12
Services aux entreprises	13
Services aux particuliers	14
Éducation, action sociale	15
Santé	16
Administration	17
Autre : (Précisez) :é é é é é é é é é é é é é é é é	18

P11. Voyez-vous encore dœanciens collègues ou dœautres personnes que vous fréquentiez dans votre vie professionnelle ?

Oui régulièrement	1
Oui de temps en temps	2
Non	3

P12. Au cours de votre vie, avez-vous connu une période de chômage de plus de 6 mois ?

Oui, une période	1
Oui, plusieurs périodes	2
Non	3

P13. Parmi les phrases suivantes, laquelle est la plus proche de votre vie professionnelle ?

Votre situation a toujours été la même	1
Votre situation s'est améliorée	2
Votre situation s'est dégradée	3
Votre situation a eu des hauts et des bas	4
Vous n'avez jamais travaillé	5
Vous êtes encore en formation	6

P14. Au cours de votre vie professionnelle, avez-vous déjà été membre d'un syndicat ou d'une organisation professionnelle ?

Oui par le passé mais plus maintenant	1
Oui encore aujourd'hui	2
Non	3

P15. Est-ce que quelque chose vous pèse dans le fait d'être à la retraite ? ✍ *Carte 2*

Oui beaucoup	1	
Oui un peu	2	
Non pas vraiment	3	➔ **P17**
Non pas du tout	4	➔ **P17**

Si oui :

P16. Qu'est-ce qui vous pèse particulièrement ? (<u>ne rien suggérer</u>)
(plusieurs réponses possibles)

Etre à la maison	1
Le manque de contacts	2
L'ennui	3
L'impression de ne pas être utile aux autres	4
L'absence de contrainte de temps	5
L'impression d'être dévalorisé aux yeux des autres	6
L'impression de perdre votre savoir-faire, vos compétences	7
Le manque d'argent, le fait de dépendre des autres financièrement	8
Autre chose : préciser ________________________________	9
Autre chose : préciser ________________________________	10
Autre chose : préciser ________________________________	11
Autre chose : préciser ________________________________	12

P17. Exercez vous une activité qui vous procure un complément de revenu ?

Oui régulièrement	1
Oui de temps en temps	2
Non	3

➔ **Aller en partie Q**

Si chômeur :

P18. Depuis quelle date êtes-vous au chômage ? **Mois |__|__| et Année |__|__|__|__|**
!!Consigne : Pour la dernière période de chômage

Si au chômage depuis plus de 6 mois :

P19. Au cours de votre vie, avez-vous connu une ou plusieurs autres périodes de chômage de plus de 6 mois ?

Oui	1
Non	2

Si au chômage depuis moins de 6 mois :

P20. Au cours de votre vie, avez-vous connu une période de chômage de plus de 6 mois ?

Oui, une période	1
Oui, plusieurs périodes	2
Non	3

P21. A la suite de quelle circonstance (principale) êtes-vous sans emploi actuellement ?
!! Consigne : ne rien suggérer

Vous avez terminé vos études	1
Vous avez terminé un stage	2
Votre contrat (CDD, intérim ou emploi occasionnel) s'est terminé	3
Vous êtes travailleur intermittent et vous vous trouvez entre deux contrats	4
Vous avez perdu votre emploi : (Précisez)	
à la suite d'un licenciement collectif, une restructuration ou fermeture de l'entreprise (y compris si vous avez dû fermer votre propre entreprise)	5
à la suite d'un licenciement individuel	6
Vous avez démissionné de votre emploi précédent ou stoppé une activité indépendante : (Précisez)	
Suite à un déménagement	7
Pour vous occuper de membres de votre famille	8
Parce que la rémunération était insuffisante	9
Parce que le travail n'était pas assez intéressant	10
Pour d'autres raisons	11
Vous avez quitté votre emploi pour des problèmes de santé personnels	12
Vous n'avez jamais travaillé ou vous ne trouvez pas de travail	13
Pour une autre raison volontaire	14
Pour une autre raison involontaire	15

P22. Etes-vous inscrit à l'ANPE ?

Oui	1
Non	2

P23. Avez-vous déjà travaillé au moins trois mois de suite ?

Oui	1	
Non	2	➔ **P28.**

Si oui :

P24. Quelle était votre profession ?
!!Consigne : celle que vous avez exercée le plus longtemps

En clairé .é é é é é é é é é é é ..
é .é é

Puis recoder : ✎ *Carte 1* |__|__|

P25. Etiez-vousé

Salarié de l'Etat	1
Salarié d'une collectivité locale, des HLM ou des hôpitaux publics	2
Salarié d'une entreprise, d'un artisan, d'une association	3
Salarié chez un particulier	4
Vous aidiez un membre de votre famille dans son travail sans être rémunéré	5
Chef d'entreprise salarié, PDG, gérant minoritaire, associé	6
Indépendant ou à votre compte	7
Autre	8

P26. Dans quel secteur d'activité était-ce ? ✎*Aide enquêteur : Carte 13*

Agriculture, sylviculture, pêche	1
Industries agricoles et alimentaires	2
Industrie des biens de consommation	3
Industrie automobile	4
Industries des biens d'équipement	5
Industries des biens intermédiaires	6
Energie	7
Construction	8

Commerce	9
Transports	10
Activités financières	11
Activités immobilières	12
Services aux entreprises	13
Services aux particuliers	14
Éducation, action sociale	15
Santé	16
Administration	17
Autre : (Précisez) :é é é é é é é é é é é é é é é é	18

P27. Voyez-vous encore dôanciens collègues ou dôautres personnes que vous fréquentiez dans votre vie professionnelle ?

Oui régulièrement	1
Oui de temps en temps	2
Non	3

P28. Etes-vous tout à fait, plutôt, plutôt pas ou pas du tout dôaccord avec les affirmations suivantes ?

✍ *Carte 3*	Tout à fait dôaccord	Plutôt dôaccord	Plutôt pas dôaccord	Pas du tout dôaccord
Ma vie familiale ou privée me gêne dans ma recherche dôemploi	1	2	3	4
Ne pas travailler pèse sur ma vie familiale ou privée	1	2	3	4
Ma vie familiale ou privée môaide dans ma situation de chômage	1	2	3	4

P29. Actuellement, recherchez-vous un emploi ?

Oui	1	
Non	2	➔ **P33.**

Si oui :

P30. Pensez-vous que vous aurez prochainement un emploi ? ✍ *Carte 4*

Oui, tout à fait	1
Oui, plutôt	2
Non, plutôt pas	3
Non, pas du tout	4

P31. Depuis un mois, pour vous aider dans vos démarches de recherche dôemploi, vous êtes-vous adresséé

	Oui une fois	Oui plusieurs fois	Non
A lôANPE, lôAPEC ?	1	2	3
A un autre organisme (mission locale, PAIO, espace jeuneé) ?	1	2	3
A la mairie ?	1	2	3

P32. Avez-vous été limité (ou empêché) dans vos démarchesé

	Oui	Non
Par le coût de la correspondance, du téléphone, des magazines spécialisés ?	1	2
Par lôabsence de moyens de transport ?	1	2
Par le coût des transports ?	1	2
Par des problèmes de santé ?	1	2
Par des problèmes de garde dôenfants (ou de personnes dont vous avez la charge) ?	1	2

➔ **Aller en P34.**

Si non :

P33. Quelle est la raison principale pour laquelle vous ne recherchez pas dêemploi actuellement ? *(ne pas citer)*

!! Consigne : plusieurs réponses possibles

Se juge trop jeune ou trop âgée pour les employeurs	1
Pense ne pas avoir la formation, les qualifications, lêexpérience requise	2
A des problèmes de santé	3
Se heurte à dêautres difficultés personnelles (discrimination, préjugés raciaux, préjugés sexistes, difficultés linguistiques, casier judiciaire)	4
Ne sait pas comment chercher	5
Pense quêil nêy a pas dêemploi à proximité	6
Pense quêil nêy a pas dêemploi dans son domaine de compétence	7
Nêa pas le droit de travailler (réfugiéé)	8
Nêest pas disponible actuellement (raisons familiales, enfants à charge, etc.)	9
Attend le résultat de démarches antérieures	10
Ne souhaite pas entreprendre de recherches immédiatement	11
Ne souhaite pas reprendre un emploi (pour le moment ou définitivement)	12
Autre raison (en clair)________________________________	13

P34. Exercez vous une activité qui vous procure un complément de revenu ?

Oui régulièrement	1
Oui de temps en temps	2
Non	3

P35. Est-ce que quelque chose vous pèse dans le fait dêêtre au chômage ? ✎ *Carte 2*

Oui beaucoup	1	
Oui un peu	2	
Non pas vraiment	3	➔ **P37**
Non pas du tout	4	➔ **P37**

Si oui :

P36. Quêest-ce qui vous pèse particulièrement ? (ne rien suggérer) (plusieurs réponses possibles)

Etre à la maison	1
Le manque de contacts	2
Lêennui	3
Lêimpression de ne pas être utile aux autres	4
Lêabsence de contrainte de temps	5
Lêimpression dêêtre dévalorisé aux yeux des autres	6
Lêimpression de perdre votre savoir-faire, vos compétences	7
Le manque dêargent, le fait de dépendre des autres financièrement	8
Autre chose : préciser ______________________________	9
Autre chose : préciser ______________________________	10
Autre chose : préciser ______________________________	11
Autre chose : préciser ______________________________	12

« Nous allons revenir sur lêensemble de votre vie professionnelleé »

P37. Parmi les phrases suivantes, laquelle est la plus proche de votre vie professionnelle ?

Votre situation a toujours été la même	1
Votre situation sêest améliorée	2
Votre situation sêest dégradée	3
Votre situation a eu des hauts et des bas	4
Vous nêavez jamais travaillé	5

Vous êtes encore en formation	6

P38. Au cours de votre vie professionnelle, avez-vous déjà été membre dôun syndicat ou dôune organisation professionnelle ?

Oui par le passé mais plus maintenant	1
Oui encore aujourdôhui	2
Non	3

➔ Aller en partie Q

Si inactif (non chômeur) :

P39. Depuis quelle date êtes-vous en inactivité ?
!! Consigne : pour la dernière période dôinactivité

Mois |__|__| et Année |__|__|__|__| si depuis toujours, coder 97

P40. A la suite de quelle circonstance (principale) êtes-vous sans emploi actuellement ?
!!Consigne : ne rien suggérer

Vous avez terminé vos études	1
Vous avez terminé un stage	2
Votre contrat (CDD, intérim ou emploi occasionnel) sôest terminé	3
Vous êtes travailleur intermittent et vous vous trouvez entre deux contrats	4
Vous avez perdu votre emploi : (Précisez)	
à la suite dôun licenciement collectif, une restructuration ou fermeture de lôentreprise (y compris si vous avez dû fermer votre propre entreprise)	5
à la suite dôun licenciement individuel	6
Vous avez démissionné de votre emploi précédent ou stoppé une activité indépendante : (Précisez)	
Suite à un déménagement	7
Pour vous occuper de membres de votre famille	8
Parce que la rémunération était insuffisante	9
Parce que le travail nôétait pas assez intéressant	10
Pour dôautres raisons	11
Vous avez quitté votre emploi pour des problèmes de santé personnels	12
Vous nôavez jamais travaillé ou vous ne trouvez pas de travail	13
Pour une autre raison volontaire	14
Pour une autre raison involontaire	15

P41. Etes-vous inscrit à lôANPE ?

Oui	1
Non	2

P42. Avez-vous déjà travaillé au moins trois mois de suite ?

Oui	1	
Non	2	**➔ P46.**

Si oui :

P43. Quelle profession avez-vous exercé le plus longtemps ?

En clairé é é é é é é é é é é é é é é é é é é .é é é é é é é é é é é ..
é .é é

Puis recoder : ☞ *Carte 1* |__|__|

P44. Etiez-vousé

Salarié de lôEtat	1
Salarié dôune collectivité locale, des HLM ou des hôpitaux publics	2
Salarié dôune entreprise, dôun artisan, dôune association	3
Salarié chez un particulier	4
Vous aidez un membre de votre famille dans son travail sans être rémunéré	5
Chef dôentreprise salarié, PDG, gérant minoritaire, associé	6
Indépendant ou à votre compte	7
Autre	8

P45. Dans quel secteur dôactivité était-ce ? *Aide enquêteur : Carte 13*

Agriculture, sylviculture, pêche	1
Industries agricoles et alimentaires	2
Industrie des biens de consommation	3
Industrie automobile	4
Industries des biens d'équipement	5
Industries des biens intermédiaires	6
Energie	7
Construction	8
Commerce	9
Transports	10
Activités financières	11
Activités immobilières	12
Services aux entreprises	13
Services aux particuliers	14
Éducation, action sociale	15
Santé	16
Administration	17
Autre : (Précisez) :é é é é é é é é é é é é é é é é	18

P46. Est-ce que quelque chose vous pèse dans le fait de ne pas avoir dôactivité professionnelle ? *Carte 2*

Oui beaucoup	1	
Oui un peu	2	
Non pas vraiment	3	➔ **P48.**
Non pas du tout	4	➔ **P48.**

<u>Si oui (réponses 1 ou 2) :</u>

P47. Quôest-ce qui vous pèse particulièrement ?

!!Consignes : ne rien suggérer, plusieurs réponses possibles

Etre à la maison	1
Le manque de contacts	2
Lôennui	3
Lôimpression de ne pas être utile aux autres	4
Lôabsence de contrainte de temps	5
Lôimpression dôêtre dévalorisé aux yeux des autres	6
Lôimpression de perdre votre savoir-faire, vos compétences	7
Le manque dôargent de dépendre des autres financièrement	8
Autre chose : préciser ________________________________	9
Autre chose : préciser ________________________________	10
Autre chose : préciser ________________________________	11
Autre chose : préciser ________________________________	12

P48. Actuellement, recherchez-vous un emploi ?

Oui	1	
Non	2	➔ **P52.**

Si oui :

P49. Pensez-vous que vous aurez prochainement un emploi ? ✍ *Carte 4*

Oui, tout à fait	1
Oui, plutôt	2
Non, plutôt pas	3
Non, pas du tout	4

P50. Depuis un mois, pour vous aider dans vos démarches de recherche dôemploi, vous êtes-vous adresséé

	Oui une fois	Oui plusieurs fois	Non
A lôANPE, lôAPEC ?	1	2	3
A un autre organisme (mission locale, PAIO, espace jeuneé) ?	1	2	3
A la mairie ?	1	2	3

P51. Avez-vous été limité (ou empêché) dans vos démarchesé

	Oui	Non
Par le coût de la correspondance, du téléphone, des magazines spécialisés ?	1	2
Par lôabsence de moyens de transport ?	1	2
Par le coût des transports ?	1	2
Par des problèmes de santé ?	1	2
Par des problèmes de garde dôenfants (ou de personnes dont vous avez la charge) ?	1	2

➔ **Aller en P53.**

Si non :

P52. Quelle est la raison principale pour laquelle vous ne recherchez pas dôemploi actuellement ?

!! Consigne : ne pas citer, plusieurs réponses possibles

Se juge trop jeune ou trop âgée pour les employeurs	1
Pense ne pas avoir la formation, les qualifications, lôexpérience requise	2
A des problèmes de santé	3
Se heurte à dôautres difficultés personnelles (discrimination, préjugés raciaux, préjugés sexistes, difficultés linguistiques, casier judiciaire)	4
Ne sait pas comment chercher	5
Pense quôil nôy a pas dôemploi à proximité	6
Pense quôil nôy a pas dôemploi dans son domaine de compétence	7
Nôa pas le droit de travailler (réfugiéé)	8
Nôest pas disponible actuellement (raisons familiales, enfants à charge, etc.)	9
Attend le résultat de démarches antérieures	10
Ne souhaite pas entreprendre de recherches immédiatement	11
Ne souhaite pas reprendre un emploi (pour le moment ou définitivement)	12
Autre raison (en clair)_______________________________	13

P53. Exercez vous une activité qui vous procure un complément de revenu ?

Oui régulièrement	1
Oui de temps en temps	2
Non	3

Si nôa jamais travaillé plus de 3 mois (P42 = 2) ➔ **Aller en partie Q.**

Si a déjà travaillé au moins 3 mois (P42 = 1) :

« Nous allons revenir sur lôensemble de votre vie professionnelleé »

P54. Au cours de votre vie, avez-vous connu une période de chômage de plus de 6 mois ?

Oui, une période	1
Oui, plusieurs périodes	2
Non	3

P55. Parmi les phrases suivantes, laquelle est la plus proche de votre vie professionnelle ?

Votre situation a toujours été la même	1
Votre situation s'est améliorée	2
Votre situation s'est dégradée	3
Votre situation a eu des hauts et des bas	4
Vous n'avez jamais travaillé	5
Vous êtes encore en formation	6

P56. Au cours de votre vie professionnelle, avez-vous déjà été membre d'un syndicat ou d'une organisation professionnelle ?

Oui par le passé mais plus maintenant	1
Oui encore aujourd'hui	2
Non	3

➔ **Aller en partie Q.**

Si actif occupé

P57. Quelle est votre profession principale actuelle ?

En clair :……………………………………….……
……………………………………….………..
Puis recoder : *Carte 1* |__|__|

P58. Dans quel secteur d'activité travaillez-vous ? *Aide enquêteur : Carte 13*

Agriculture, sylviculture, pêche	1
Industries agricoles et alimentaires	2
Industrie des biens de consommation	3
Industrie automobile	4
Industries des biens d'équipement	5
Industries des biens intermédiaires	6
Energie	7
Construction	8
Commerce	9
Transports	10
Activités financières	11
Activités immobilières	12
Services aux entreprises	13
Services aux particuliers	14
Éducation, action sociale	15
Santé	16
Administration	17
Autre : (Précisez) :………………	18

P59. Exercez-vous votre profession…

A votre compte	1	➔ **P62**
Comme salarié	2	
Vous aidez un membre de votre famille dans son travail, sans être salarié	3	➔ **P62**
Sans contrat de travail	4	➔ **P62**

Si salarié :

P60. Etes-vous…

Salarié de l'Etat	1
Salarié d'une collectivité locale, des HLM ou des hôpitaux publics	2
Salarié d'une entreprise, d'un artisan, d'une association	3
Salarié chez un particulier	4
Vous aidiez un membre de votre famille dans son travail sans être rémunéré	5

Chef dôentreprise salarié, PDG, gérant minoritaire, associé	6
Autre	7

P61. Quel type de contrat avez-vous ?

Apprentissage sous contrat	1
Stage rémunéré en entreprise (y compris stage dôentrée dans la profession pour les avocats stagiaires, etc.)	2
En période dôessai	3
Emploi jeune, CES, contrat de qualification ou autre emploi aidé	4
Autre emploi à durée limitée, CDD, contrat court, saisonnier, vacataire, etc.	5
Emploi sans limite de durée, CDI, titulaire de la Fonction publique	6

Enquêteur

P62 Situation professionnelle (*A remplir directement, dôaprès les réponses aux questions précédentes ou en demandant un complément dôinformation) :*

1. A une activité professionnelle effective (y compris apprentissage, stage, congé-maternité, congé-maladie)
2. Elève fonctionnaire
3. Stagiaire en formation (FPA, stage de préparation à lôemploi, stage FNE ou modulaire pour chômeur de longue durée)
4. En arrêt de travail de plus dôun an (longue maladie, accident) ➔ **P78.**
5. En dispense dôactivité ou en congé de conversion ➔ **P78.**
6. En congé parental ➔ **P78.**

P63. Combien de personnes travaillent sur votre lieu de travail ?

Vous travaillez totalement seul(e)	1
Moins de 10 personnes	2
De 10 à 24 personnes	3
De 25 à 49 personnes	4
De 50 à 99 personnes	5
De 100 personnes à 499 personnes	6
500 personnes ou plus	7
Ne sait pas	8

P64. Combien dôheures par semaine travaillez-vous habituellement dans votre emploi, en comptant les heures supplémentaires ?

!!Consigne : il sôagit du temps réel travaillé, et non du temps prévu par lôéventuel contrat de travail. Prenez en compte les heures supplémentaires. Ne prenez pas en compte les heures de repas. Si le nombre dôheures varie, indiquez la moyenne au cours des 4 dernières semaines.

[___|___|___] heures

<u>Si moins de 32 heures par semaine :</u>

P65. Quelle est la principale raison qui vous amène à travailler à temps partiel ?

Parce que vous nôavez pas trouvé dôemploi à plein temps	1
Pour poursuivre une formation, des études	3
Pour raisons de santé (maladie, handicap)	2
Pour vous occuper dôun enfant ou dôautres personnes	4
Pour dôautres raisons volontaires	5
Pour dôautres raisons involontaires	6

P66. Concernant vos horaires de travailé

	Tout le temps	Souvent ou en alternance (par ex les « 3-8 »)	Parfois	Jamais
Travaillez-vous de nuit (entre minuit et 5 heures du matin)é ?	1	2	3	4
Avez-vous des horaires fractionnés (une interruption de 3 heures ou plus dans votre journée de travail)é ?	1	2	3	4
Avez-vous des horaires tournants (qui changent dôune semaine sur lôautre par exemple) ?	1	2	3	4

P67. Combien de temps de trajet mettez-vous habituellement aller et retour, entre votre domicile et votre lieu de travail ?
!! Attention : temps total aller et retour (si plusieurs lieux de travail, prendre le plus courant ; si pas de trajet habituel, coder 999 ; si travail à domicile, coder 000)

|___|___|___| minutes

P68. Quel moyen de transport utilisez-vous en général pour vous rendre sur votre lieu de travail ?
!!Consigne : plusieurs réponses possibles

Pas de transport, travaille à domicile	1
Marche à pieds	2
Vélo	3
Véhicule motorisé (voiture, moto, scooter), y compris en co-voiturage	4
Transports en commun	5
Trop variable pour répondre	6

P69. Où se trouve votre lieu de travail habituel ?

A Paris intra-muros	1	préciser la station de métro : ___________________________
Hors Paris	2	préciser la commune : ________________________________
Itinérant	3	
A domicile	4	
(trop variable pour répondre)	5	

P70. Dans votre travail, êtes-vous satisfait deé

✍ Carte 14	Très satisfait	Plutôt satisfait	Plutôt pas satisfait	Pas du tout satisfait	Sans objet
La rémunération (paye, salaire) ?	1	2	3	4	
Les perspectives de promotion ?	1	2	3	4	
La possibilité dûtiliser vos compétences ?	1	2	3	4	
Les possibilités dônnovation et de créativité ?	1	2	3	4	
La possibilité dâtteindre vos objectifs professionnels ?	1	2	3	4	
La formation offerte ?	1	2	3	4	
Les avantages sociaux ?	1	2	3	4	
Les relations avec vos collègues ?	1	2	3	4	5
Les relations avec vos supérieurs ?	1	2	3	4	5
La durée de votre temps de travail ?	1	2	3	4	
Vos horaires de travail ?	1	2	3	4	
Votre sécurité dêmploi ?	1	2	3	4	
La capacité de contribuer à quelque chose dûtile pour la société ?	1	2	3	4	

P71. A quelle fréquenceé

✍ Carte 15	Très souvent	Assez souvent	Rarement	Jamais
Trouvez-vous votre travail stressant ?	1	2	3	4
Rentrez-vous du travail très fatigué ?	1	2	3	4
Continuez-vous à vous soucier des problèmes rencontrés dans votre travail après avoir quitté votre lieu de travail ?	1	2	3	4
Vous avez votre mot à dire concernant votre travail ?	1	2	3	4
Votre travail est intéressant ?	1	2	3	4
Vous pouvez prendre des initiatives dans votre travail ?	1	2	3	4
Avez-vous le sentiment de ne pas pouvoir réaliser correctement votre travail faute de temps ?	1	2	3	4
Vous ennuyez-vous dans votre travail ?	1	2	3	4
Votre travail est varié ?	1	2	3	4

P72. Voici une liste dôaffirmations relative à votre travail actuel. Pour chacune dôelles, diriez-vous que vous êtes tout à fait dôaccord, plutôt dôaccord, plutôt pas dôaccord ou pas du tout dôaccord?

✍ *Carte 3*	Tout à fait dôaccord	Plutôt dôaccord	Plutôt pas dôaccord	Pas du tout dôaccord	Sans objet
Vous recevez dans votre travail tout le respect et lôestime que vous méritez	1	2	3	4	
Vous recevez beaucoup de soutien de votre hiérarchie quand il y a de la pression au travail	1	2	3	4	5
Votre entreprise ou organisation fait face à des difficultés financières en ce moment	1	2	3	4	5
Vous arrivez bien à concilier votre vie professionnelle avec votre vie familiale	1	2	3	4	5
Vous êtes prêt à travailler plus que vous ne le devez afin dôaider votre entreprise (ou votre organisation) à réussir	1	2	3	4	
Vous êtes fier de travailler pour votre entreprise (ou votre organisation)	1	2	3	4	

P73. Pensez-vous que votre travail correspond à votre qualification, ou vos capacités ?

Côest moins qualifié que ce que vous savez faire	1
Cela correspond à votre qualification ou vos capacités	2
Côest plus qualifié que ce que vous aviez appris ou fait auparavant	3

Si la personne est à son compte :

P74. Pensez vous que dans les deux prochaines années votre entreprise risque de connaître des problèmes :

Sans objet (la personne compte prendre sa retraite dôci là ou quitter volontairement son entreprise)	1
Sans doute que oui	2
Peut être que oui	3
Probablement non	4
Ne sait vraiment pas	5

Si salarié :

P75. Pensez vous que dans les deux prochaines années il y ait un risque que vous soyez licencié (collectivement ou individuellement) ?

Sans objet (la personne compte prendre sa retraite dôci là ou quitter volontairement son entreprise)	1
Sans doute que oui	2
Peut être que oui	3
Probablement non	4
Ne sait vraiment pas	5

P76. En plus de votre activité principale, exercez-vous un autre emploi, de quelque type que ce soit (salarié, indépendant, etc.) é

De façon régulière, à longueur dôannée	1	
De façon saisonnière	2	➔ **P78.**
De façon occasionnelle	3	➔ **P78.**
De façon exceptionnelle	4	➔ **P78.**
Jamais	5	➔ **P78.**

Si 1 (occupe un autre emploi de façon régulière) :

P77. Combien dôheures par mois consacrez-vous à cette autre activité ?

[____|____|____] heures

P78 Au cours de votre vie, avez-vous connu une période de chômage de plus de 6 mois ?

Oui, une période	1
Oui, plusieurs périodes	2
Non	3

P79. Parmi les phrases suivantes, laquelle est la plus proche de votre vie professionnelle ?

Votre situation a toujours été la même	1
Votre situation sœst améliorée	2
Votre situation sœst dégradée	3
Votre situation a eu des hauts et des bas	4
Vous êtes encore en formation	5

P80. Au cours de votre vie professionnelle, avez-vous déjà été membre dœun syndicat ou dœune organisation professionnelle ?

Oui par le passé mais plus maintenant	1
Oui encore aujourdœhui	2
Non	3

Q- RESSOURCES MATERIELLES ET FINANCIERES

« Nous allons maintenant parler de quelques ressources matérielles et financières »

Q1. En résumant votre situation financière, depuis que vous êtes adulte, avez-vous connu des périodes difficiles ? *(1 seule réponse possible)*

Vous nœen avez jamais eu	1
Vous en avez eu par le passé mais plus maintenant	2
Vous en avez depuis un certain temps	3
Vous en avez toujours eu de façon périodique	4
Vous en avez toujours eu	5

Q2. Etant donné les revenus de votre ménage, actuellement, diriez-vous que financièrementé

Vous êtes à lœaise	1
Ça va	2
Cœst juste, il faut faire attention	3
Vous y arrivez difficilement	4

Q3. Compte tenu de votre niveau de vie et celui des ménages dœIle-de-France, vous estimez faire partieé

Des 20% les plus pauvres	1
Des 20% moyennement pauvres	2
Des 20% qui sont au milieu	3
Des 20% moyennement riches	4
Des 20% les plus riches	5

Q4. Diriez vous que les revenus de votre ménage sont actuellement :

Stables	1
A peu près stables	2
Instables	3

Q5. A votre avis, quel serait le revenu net mensuel le plus bas que votre foyer devrait avoir, afin de joindre les deux bouts, étant données les circonstances présentes et la composition de votre foyer ?

!! Consigne : par revenu net on entend le revenu après que les impôts et les charges sociales ont été déduits.

|___|___|___|___|___| ú / mois

Si ne sait pas : coder 99999
Si refus de répondre, coder 88888

Q6. Quelles sont actuellement les sources et le montant mensuel des revenus de votre ménage ? Votre ménage perçoit-ilé

	Oui	Non	*NSP*	Montant net en ú (si NSP exactement, indiquez une approximation)
Les revenus du travail de vous-même ou des autres membres du ménage ?	1	2	*3*	
Les rémunérations de stage de formation ?	1	2	*3*	
Les Assurances chômage ?	1	2	*3*	
Lôallocation spécifique de solidarité (chômeur en fin de droit) ?	1	2	*3*	
Le RMI ?	1	2	*3*	
Les allocations familiales ?	1	2	*3*	
Les allocations parent isolé (API) ?	1	2	*3*	
Les Pensions alimentaires ?	1	2	*3*	
La retraite, préretraite ?	1	2	*3*	
Le minimum vieillesse ?	1	2	*3*	
Les allocations aux adultes handicapés (AAH) ?	1	2	*3*	
Les aides logement (APL, ALS, ALF, FSL) ?	1	2	*3*	
Lôaide financière donnée par des services sociaux ?	1	2	*3*	
Lôaide financière donnée par la famille (ou des proches) ?	1	2	*3*	
Les « petits boulots » ?	1	2	*3*	
Les revenus financiers tels que loyers, placements dôargent ?	1	2	*3*	
Autres sources : précisez _____________________________	1	2	*3*	

Q7. Donc au total votre ménage dispose par mois de :
***!!** Enquêteur : faire la somme*

En clair	_____________________________
(En tranche : montrer la carte)	_____________________________
(Ne sait pas)	99
(Ne veut pas répondre)	98

Q8. Aujourdôhui, votre ménage a-t-il des dettes ou des emprunts à rembourser ?

Oui	1	
Non	2	➔ **Q10**

Si oui :

Q9. Ces dettes et ces empruntsé

Ne posent pas de difficultés à votre ménage	1
Vous posent des difficultés que vous surmontez	2
Vous posent des difficultés au point de ne pas pouvoir rembourser ou de devoir ré-emprunter pour rembourser	3

Q10. Votre foyer dispose-t-il dôun ou plusieurs biens suivants ?

	Oui	Non
Téléphone (fixe ou mobile)	1	2
Ordinateur (portable ou non)	1	2
Connexion à Internet	1	2
Voiture ou deux-roues à moteur	1	2

Q11. Vous-même, avez-vous un permis de conduire valide ?

Oui	1
Non	2

Q12. Vous êtes-vous déjà connecté personnellement à Internet ? *(Plusieurs réponses possibles)*

	Oui régulièrement	Oui occasionnellement	Non
A votre domicile	1	2	3
Sur votre lieu de travail (ou dôétude)	1	2	3
Chez des amis ou des membres de votre famille	1	2	3
Dans un lieu public (cyber-café, bibliothèque, bureau de poste, centre commercial, point jeune, centre dôaction socialeé)	1	2	3

Si aucun « oui » : ➔ ***aller en R1.***
Si au moins un « oui » (occasionnellement ou régulièrement) :

Q13. Depuis combien de temps utilisez-vous Internet ?

Moins de 6 mois	1
Entre 6 et 12 mois	2
Entre 1 et 3 ans	3
Entre 4 et 6 ans	4
Depuis plus de 7 ans	5

Q14. En dehors de la messagerie électronique / email, à quelle fréquence vous connectez-vous à Internet ?

Tous les jours	1
Une à deux fois par semaine	2
Une à deux fois par mois	3
Plus rarement	4

R. COUPLE ET FAMILLE

« A présent, nous allons aborder certaines questions liées à la vie conjugale et familiale »

R1. Avez-vous eu ï vous personnellement ï des enfants, quõil sõagisse de vos enfants biologiques ou dõenfants adoptés ?

Oui	1	
Non mais vous en attendez un	2	➔ **R8.**
Non	3	➔ **R8.**

Si oui :

**R2. En commençant par le plus âgé, pouvez-vous me donner leur année de naissance ?** Année de naissance *(YYYY)*	**Lõun ou plusieurs dõentre eux sont-ils aujourdõhui décédés ?** **Si oui, lesquels ?** *cocher*	**(Concernant ceux qui sont encore vivants,) où vivent-ils aujourdõhui ?** 1. avec vous et son autre parent 2. avec vous 3. avec son autre parent 4. en alternance chez vous et son autre parent 5. placé en foyer, famille dõaccueil 6. chez dõautres personnes 7. dans une autre structure (pensionnat, hôpitalé) 8. indépendant 9. autre
1er enfant né |__|__|__|__|	☐	|___|
2ème |__|__|__|__|	☐	|___|
3ème |__|__|__|__|	☐	|___|
4ème |__|__|__|__|	☐	|___|
5ème |__|__|__|__|	☐	|___|
6ème |__|__|__|__|	☐	|___|
7ème |__|__|__|__|	☐	|___|
8ème |__|__|__|__|	☐	|___|
9ème |__|__|__|__|	☐	|___|
10ème |__|__|__|__|	☐	|___|
11ème |__|__|__|__|	☐	|___|
12ème |__|__|__|__|	☐	|___|
13ème |__|__|__|__|	☐	|___|
14ème |__|__|__|__|	☐	|___|
15ème |__|__|__|__|	☐	|___|

R3. Au cours de votre vie, lôun au moins de vos enfants a-t-il été placé (par la DDASS, la PJJ, lôASE)?

Oui	1
Non	2

<u>Pour les personnes qui ont des enfants de plus de 6 ans</u> : ***(les autres : ➔ R6.)***

R4. Parmi vos enfants, y en a-t-il avec lesquels vous avez :

	Oui	Non
De bonnes relations	1	2
De mauvaises relations	1	2
Aucune relation *(pour des raisons autres que décès, disparitioné)*	1	2

R5. Avez-vous le sentiment de compter pour vos enfants ?

Oui pour tous	1
Oui pour certains	2
Non pour aucun	3

<u>Pour les personnes qui ont des enfants ne vivant pas avec elles</u> : ***(les autres : ➔ R8.)***

R6. <u>Concernant les enfants qui ne vivent pas avec vous</u> : A quelle fréquence avez vous des contacts avec lôun ou lôautre de vos enfants, que ce soit des rencontres ou contacts par téléphone ?

Rencontres (contacts « physiques »)		Contacts téléphoniques, courrier, mails, etc.	
|____| fois par	1- jour 2- semaine<br>3- mois 4- an	|____| fois par	1- jour 2- semaine<br>3- mois 4- an
< 1 fois par an	5	< 1 fois par an	5
Jamais	6	Jamais	6

R7. Aimeriez vous avoir des contacts avec eux : *(plusieurs réponses possibles)*

Plus souvent	1
Côest juste ce quôil faut	2
Moins souvent	3
Ne sait pas	4

<u>A tous :</u>

« Nous allons revenir dans votre passé »

R8. Que vous ayez ou non été marié, avez-vous vécu par le passé une rupture de vie de couple ? *(plusieurs réponses possibles)*
!! Consigne : pas toutes les séparations sentimentales
!! Consigne : lire tous les items

Oui, une séparation ou un divorce	1	
Oui, le décès de votre conjoint	2	➔ **R13.**
Non, mais éloignement géographique de longue durée (plus dôun an)	3	➔ **R13.**
Non	4	➔ **R13.**

<u>Si a vécu une séparation ou un divorce (1) :</u>

R9. Combien de ruptures de ce type (ou ces types) avez vous vécu ?

Nombre |___|___|

R10. A quelle date remonte-t-elle / A quelle date remonte la dernière ?

|___|___| |___|___|___|___|
mois année

R11. Si vous deviez faire le bilan de cette (dernière) rupture, diriez-vous que cette rupture a été *(une seule réponse possible)*

Une souffrance	1	
Un soulagement	2	➔ **R13.**
Un peu des deux	3	

Si 1 ou 3 (souffrance ou un peu des deux) :

R12. En souffrez-vous toujours aujourd'hui ?

Oui, beaucoup	1
Oui, un peu	2
Non	3

Pour tous ceux qui ont vécu en couple au moins une fois ou connaissent une relation sentimentale (sinon : ➔ S1.)

R13. Au cours de votre (ou vos) vie(s) de couple, avez-vous vécu les événements suivants :

	Oui actuellement	Oui auparavant	Non	Sans objet
Maladie grave de votre partenaire	1	2	3	
Comportements violents de votre conjoint à votre égard	1	2	3	
Comportements violents de votre part à l'égard de votre conjoint	1	2	3	
Alcoolisme ou toxicomanie de la part de votre conjoint	1	2	3	
Si a eu des enfants :				
Comportements violents de votre conjoint à l'égard de vos enfants	1	2	3	4
Comportements violents de votre part à l'égard de vos enfants	1	2	3	4
Comportements violents de l'un de vos enfants envers vous-même	1	2	3	4

R14. Avez-vous dans votre vie de couple actuelle, le sentiment que votre conjoint vous dévalorise ou vous rabaisse ?

En permanence	1
Parfois	2
Jamais	3

Si a connu une autre vie de couple auparavant :

R15 Et dans vos précédentes vies de couple, aviez-vous eu le sentiment que votre conjoint vous dévalorisait ou vous rabaissait ?

En permanence	1
Parfois	2
Jamais	3

R16. Actuellement, vivez-vous en cohabitation (que vous soyez marié ou non) ?

Oui	1	➔ **depuis quelle année ?** |___|___|___|___|	➔ **R18.**
Non	2		

Si non : **R17. Avez-vous actuellement une relation sentimentale ou amoureuse qui est importante pour vous ?**

Oui	1	
Non	2	➔ **S1.**

R18. Aujourd'hui, avez vous l'impression de compter pour votre conjoint ou la personne avec qui vous entretenez une relation ? ✎ *Carte 2*

Oui beaucoup	1
Oui un peu	2
Non pas beaucoup	3
Non pas du tout	4
(Ne sait vraiment pas)	*5*

S. RELATIONS AVEC LES AUTRES

« Nous allons passer à des questions portant sur vos amis et la vie sociale en général »

S1. Combien avez-vous dôamis ou de relations amicales (en dehors des personnes de votre famille) ? *(citer les réponses)*

Aucun	0	➔ **S5.**
1	1	
De 2 à 4	2	
De 5 à 9	3	
10 ou plus	4	

<u>Si au moins un ami :</u>

S2. A quelle fréquence avez vous des contacts avec lôun ou lôautre de vos amis ?

Rencontres (contacts « physiques »)		Contacts téléphoniques, courrier, mails, etc.	
|____| fois par	1- jour 2- semaine<br>3- mois 4- an	|____| fois par	1- jour 2- semaine<br>3- mois 4- an
< 1 fois par an	5	< 1 fois par an	5
Jamais	6	Jamais	6

S3. Aimeriez vous avoir des contacts avec eux (lui/elle)é
!! Plusieurs réponses possibles

Plus souvent	1
Côest juste ce quôil faut	2
Moins souvent	3
(ne pas citer) Ne sait pas	4

S4. Parmi vos amis (hors membres du ménages), certains habitent-ilsé

	Oui, la plupart (plus de la moitié)	Oui, certains (moins de la moitié)	Non, aucun
Dans le même quartier que vous ?	1	2	3
Hors du quartier mais dans la même commune (arrondissement pour Paris) que vous ?	1	2	3

<u>A tous :</u>

S5. En cas de besoin, est-ce que vous pourriez compter sur quelquôun pouré ✎ ***Carte 16***
!! Consigne : si non concerné, coder « Non »

		<u>Si oui, pourriez-vous compter sur desé</u>				
		Membres du ménage	Enfants / parents (hors ménage)	Autres membres de la famille	Amis, collègues	Voisins
Vous aider à trouver du travail ?	1. oui<br>2. non	1. oui<br>2. non	1. oui<br>2. non	1. oui<br>2. non	1. oui<br>2. non	1. oui<br>2. non
Vous aider à trouver un logement ?	1. oui<br>2. non	1. oui<br>2. non	1. oui<br>2. non	1. oui<br>2. non	1. oui<br>2. non	1. oui<br>2. non
Vous aider financièrement ou matériellement (nourriture, vêtements, etc.) ?	1. oui<br>2. non	1. oui<br>2. non	1. oui<br>2. non	1. oui<br>2. non	1. oui<br>2. non	1. oui<br>2. non
Vous aider dans la vie quotidienne, vous donner un coup de main (*)é ?	1. oui<br>2. non	1. oui<br>2. non	1. oui<br>2. non	1. oui<br>2. non	1. oui<br>2. non	1. oui<br>2. non
Vous apporter un soutien moral ou affectif ?	1. oui<br>2. non	1. oui<br>2. non	1. oui<br>2. non	1. oui<br>2. non	1. oui<br>2. non	1. oui<br>2. non

** Enq : vous aider à vous rendre quelque part, pour quelques menus travaux chez vous, garder vos enfants, etc.*

S6. Au cours des six derniers mois, avez-vous été aidéé *Carte 17*

		Si oui, était-ce par...			
		Enfants, parents	Autres membres de la famille	Amis, collègues	Voisins
Financièrement ou matériellement (nourriture, vêtements, etc.) ?	1. oui<br>2. non	1. oui<br>2. non	1. oui<br>2. non	1. oui<br>2. non	1. oui<br>2. non

Si non, avez-vous eu pourtant besoin de ce type dôaide ? 1. oui 2. non

Dans la vie quotidienne, pour un coup de mainé ?	1. oui<br>2. non	1. oui<br>2. non	1. oui<br>2. non	1. oui<br>2. non	1. oui<br>2. non

Si non, avez-vous eu pourtant besoin de ce type dôaide ? 1. oui 2. non

Pour un soutien moral ou affectif ?	1. oui<br>2. non	1. oui<br>2. non	1. oui<br>2. non	1. oui<br>2. non	1. oui<br>2. non

Si non, avez-vous eu pourtant besoin de ce type dôaide ? 1. oui 2. non

S7. Au cours des 6 derniers mois, avez-vous aidé ou aidez-vous actuellement quelquôuné *Carte 17*

		Si oui, avez-vous aidé ou aidez-vous des...			
		Enfants, parents	Autres membres de la famille	Amis, collègues	Voisins
Financièrement ou matériellement (nourriture, vêtements, etc.) ?	1. oui<br>2. non	1. oui<br>2. non	1. oui<br>2. non	1. oui<br>2. non	1. oui<br>2. non
Dans la vie quotidienne, pour un coup de mainé ?	1. oui<br>2. non	1. oui<br>2. non	1. oui<br>2. non	1. oui<br>2. non	1. oui<br>2. non
Pour un soutien moral ou affectif ?	1. oui<br>2. non	1. oui<br>2. non	1. oui<br>2. non	1. oui<br>2. non	1. oui<br>2. non

S8. Participez-vous aux activités dôune ou plusieurs associations (culturelle, politique, de parents dôélèves, dôanimation ou défense du quartier, etc.) ?
!! Consigne : sans compter les dons faits à des associations

Oui	1	
Non	2	➔ **S12.**

Si oui : ***S9. Au total, à combien dôassociations participez-vous ? |___|___| associations***

S10. Combien sont situées dans votre quartier ? **|___|___|** dans mon quartier

S11. Parmi les associations auxquelles vous participez, y en a-t-il dans lesquellesé

	Oui	Non
é vous exercer des activités bénévoles ?	1	2
é vous avez une responsabilité ?	1	2

S12. Avez-vous le sentiment dôappartenir à une « communauté » ethnique ou religieuse ?

Oui	1
Non	2

S13. Avez-vous le sentiment dôappartenir à un autre type de « communauté » ?

Oui	1
Non	2

S14. Vis-à-vis de la religion, avez-vousé

Une pratique religieuse régulière	1
Une pratique religieuse occasionnelle	2
Pas de pratique mais le sentiment dôappartenir à une religion	3
Ni pratique ni sentiment dôappartenance	4

S15. En dehors des activités professionnelles, les activités de tous les jours peuvent comprendre dœautres tâches, comme sœoccuper dœautres personnes.
Actuellement, vos activités quotidiennes comprennent-elles des soins apportés à une ou plusieurs personne malade, handicapée ou âgée (vivant ou non dans votre ménage) sans que vous soyez rémunéré ?

Oui	1	
Non	2	➔ **S17.**

Si oui :

S16. Combien de temps par jour cela vous prend-il ?
!! Consigne : si cela nœst pas tous les jours, faire une moyenne sur la semaine et diviser par 7

|____|____|____| minutes

S17. Dœune façon générale, vous diriez que vous vous sentez très seul, plutôt seul, plutôt entouré ou très entouré ?

Très seul	1
Plutôt seul	2
Plutôt entouré	3
Très entouré	4

S18. Votre situation se rapproche-t-elle des expressions suivantes ?

✎ *Carte 15*	Très souvent	Assez souvent	Rarement	Jamais
Vous vous sentez sous pression	1	2	3	4
Vous prenez les choses du bon côté	1	2	3	4
Vous avez confiance en vous	1	2	3	4
Vous vous sentez bien dans votre peau	1	2	3	4
Vous vous sentez rejeté(e) par les autres	1	2	3	4
Certaines personnes vous jugent négativement à cause de vos revenus ou de votre situation professionnelle	1	2	3	4

S19. Au cours des 12 derniers mois, êtes-vous parti en vacances ? (Cœst-à-dire hors de votre domicile habituel pour au moins 4 jours consécutifs)

Oui	1
Non	2

T. RAPPORT AUX INSTITUTIONS

« Pour conclure je vais vous poser quelques questions relatives à votre opinion sur le fonctionnement des institutions »

T1. Estimez-vous que vous êtes suffisamment informé de tous vos droits ? ✎ *Carte 4*

Oui, tout à fait	1
Oui plutôt	2
Non, plutôt pas	3
Non, pas du tout	4
(Ne pas citer) Ne sait pas	5

T2. Dans la société actuelle, pensez-vous que vos propres droits sont respectés ? ✎ *Carte 4*

Oui, tout à fait	1
Oui plutôt	2
Non, plutôt pas	3
Non, pas du tout	4
(Ne pas citer) Ne sait pas	5

T3. Etes-vous satisfait de la façon dont la démocratie fonctionne en France ? ☞ *Carte 4*

Oui, tout à fait	1
Oui plutôt	2
Non, plutôt pas	3
Non, pas du tout	4
(Ne pas citer) Ne sait pas	5

T4. Quelle est votre nationalité <u>actuelle</u> ? *(plusieurs réponses possibles)*

Français	1
Autre : en clair ______________________________	2
Autre : en clair ______________________________	3
Autre : en clair ______________________________	4

<u>Si nœst pas Français(e) :</u>

T5. Actuellement, êtes-vous en possession deé (*Citer*)

Une carte de résident de 10 ans avec droit de travailler ?	1
Une carte de séjour de moins de 10 ans avec droit de travailler ?	2
Une carte de séjour de moins de 10 ans sans droit de travailler ?	3
Du statut de réfugié ?	4
De documents prouvant que vous êtes demandeur dœasile ?	5
Une carte de séjour pour soins ?	6
Sans papier ?	7
(ne pas citer) Ne souhaite pas répondre ?	*8*

➔ **Aller en T9.**

<u>Si Français :</u>

T6. Avez-vous voté lors de la dernière élection présidentielle en 2002 ?

Oui	1	➔**T8**
Non	2	

<u>Si non :</u>

T7. Pour quelle raison ?

Vous nœtiez pas inscrit sur les listes électorales	1
Il nœy avait pas de candidat représentant vos aspirations	2
Voter ne sert jamais à rien	3
Autre raison	4

T8. Avez-vous voté lors du dernier référendum sur le traité constitutionnel européen ?

Oui	1
Non	2

T9. Actuellement, êtes-vous inscrit pour voter sur des listes électoralesé *(plusieurs réponses possibles)*

Oui, en France	1
Oui, dans un autre pays	2
Non, dans aucun pays	3

T10. Vous-même, avez-vous déjà été élué :

	Oui	Non
Dans le cadre de vos études ?	1	2
Dans le cadre de votre travail ?	1	2
Dans le cadre de la vie politique (locale ou nationale) ?	1	2
Dans un autre cadre ?	1	2

T11. Au cours des cinq dernières années, avez-vous participé à des mouvements, manifestations, grèves, ou signé des pétitions ?

Oui	1
Non	2

T12. Vous sentez vous attaché aux principes de la République française ? ✎ *Carte 18*

Oui fortement	1
Oui un peu	2
Non plutôt pas	3
Non pas du tout	4
(Ne pas citer) Ne sait pas	*5*

T13. Vous sentez vous proche dún parti politique ?

Oui	1
Non	2

T14. Personnellement, avez-vous confiance dansé

	Oui, très confiance	Oui, plutôt confiance	Non, plutôt pas confiance	Non, pas du tout confiance
Le système judiciaire ?	1	2	3	4
La police ?	1	2	3	4
Le système de santé ?	1	2	3	4
LóEducation nationale (le système dóéducation public) ?	1	2	3	4
Le système de protection sociale ?	1	2	3	4
Les média (presse écrite, radio, TV) ?	1	2	3	4
Les partis politiques ?	1	2	3	4
Les syndicats ?	1	2	3	4
Les institutions politiques nationales (Gouvernement, Assemblée Nationale, Sénat) ?	1	2	3	4

T15. Au cours de lóannée écoulée, dans les différents domaines de votre vie personnelle et sociale, avez-vous été personnellement victime deé ?

	Oui souvent	Oui de temps en temps	Non
Racisme (discrimination liée à votre couleur, origine géographique, etc.) ?	1	2	3
Discrimination liée à votre lieu de résidence ?	1	2	3
Discrimination liée à votre situation sociale ou financière ?	1	2	3
Discrimination liée au fait dóêtre un homme ou une femme ?	1	2	3
Discrimination liée à vos opinions ou vos activités politiques ou syndicales ?	1	2	3
Discrimination liée à votre religion ?	1	2	3
Discrimination liée à votre état de santé ou handicaps que vous auriez ?	1	2	3
Autre type de discrimination ?	1	2	3

Si au moins un oui :

T16. Où, ou dans quelle occasion ?

	Oui	Non
Au travail ou en cherchant un emploi	1	2
Dans une institution publique (administration, école, hôpital, etc.)	1	2
Dans le cadre de la famille, ou dóaffaires familiales	1	2
Dans le cadre de relations amicales	1	2
Dans le cadre du voisinage	1	2
Dans une autre situation : en clair ______________________________	1	2

T17. Pensez-vous que la société devrait êtreé

Radicalement changée ?	1
Réformée pour lóessentiel ?	2
Réformée sur plusieurs points sans changer lóessentiel ?	3
Laissée en lóétat actuel ?	4

T18. Etes-vous tout à fait dôaccord, plutôt dôaccord, plutôt pas dôaccord ou pas dôaccord du tout avec les propositions suivantes :

✍ *Carte 3*	Tout à fait dôaccord	Plutôt dôaccord	Plutôt pas dôaccord	Pas du tout dôaccord	*(Ne sait vraiment pas)*
C'est la responsabilité du gouvernement de réduire les écarts entre ceux qui ont un revenu élevé et ceux qui ont un faible revenu	1	2	3	4	*5*
Le gouvernement devrait garantir que tous les enfants aient un niveau de vie décent	1	2	3	4	*5*
Le gouvernement devrait dépenser <u>moins</u> en allocations ou en avantages destinés aux pauvres	1	2	3	4	*5*
Le gouvernement devrait assurer à chacun un revenu minimum garanti	1	2	3	4	*5*
Le gouvernement devrait assurer un logement décent à tous ceux qui n'en ont pas les moyens	1	2	3	4	*5*
Le gouvernement devrait fournir un travail à toute personne qui en veut un	1	2	3	4	*5*
Le gouvernement devrait assurer un niveau de vie acceptable aux personnes sans emploi	1	2	3	4	*5*
Vous seriez prêt à payer plus dôimpôts si on était certain quôils soient utilisés pour améliorer la situation des pauvres	1	2	3	4	*5*

Merci dôavoir répondu à cette enquête.

Pour vérifier le travail des enquêteurs, pour vous communiquer les résultats de cette étude, et pour pouvoir éventuellement vous recontacter dans lôavenir, merci de nous communiquer vos coordonnées.

Les conditions relatives à la loi Informatique et Libertés (CNIL) vous sont rappelées sur la lettre qui vous a été remise au début de lôentretien, notamment votre droit dôaccès et de rectification au fichier nominatif.

!! Consigne : écrire lisiblement SVP

Vos coordonnées

Nom ____
Prénom ____
Adresse 1 ____
Adresse 2 ____
Code postal |__|__|__|__|__|
Ville ____

Téléphone fixe |__|__|__|__|__|__|__|__|__|__|
Téléphone portable |_0|_6|__|__|__|__|__|__|__|__|
Téléphone professionnel |__|__|__|__|__|__|__|__|__|__| Sôagit-il dôune ligne directe ? *1* oui *2* non *3* nsp
Adresse email ______________________@_____________________

Sôagit-il dôune adresse
1 personnelle individuelle
2 personnelle collective
3 professionnelle individuelle
4 professionnelle collective
5 (NSP)

Pour pouvoir approfondir nos résultats dans le futur, nous souhaiterions pouvoir vous recontacter dans les mois ou années qui viennent. Pour cette raison et si vous étiez amené(e) à déménager, nous aimerions avoir les coordonnées de personnes qui vous sont proches et à qui nous pourrions demander votre nouvelle adresse.

<u>Ces personnes ne seront pas invitées à participer à lôenquête et aucun résultat ne leur sera communiqué</u>. <u>Il leur sera juste demandé, la cas échéant, vos nouvelles coordonnées</u>.

Contact 1

Nom ______________________ Prénom ______________________
Liens avec vous *1* Conjoint *2* Parent direct *3* Enfant direct *4* Ami *5* Autre famille *6* Autre
Si 5 ou 6 : précisez en clair ____
Téléphone fixe |__|__|__|__|__|__|__|__|__|__|
Téléphone portable |_0|_6|__|__|__|__|__|__|__|__|
Téléphone professionnel |__|__|__|__|__|__|__|__|__|__|
Adresse email _____________________@__________________

Contact 2

Nom ______________________ Prénom ______________________
Liens avec vous *1* Conjoint *2* Parent direct *3* Enfant direct *4* Ami *5* Autre famille *6* Autre
Si 5 ou 6 : précisez en clair ____
Téléphone fixe |__|__|__|__|__|__|__|__|__|__|
Téléphone portable |_0|_6|__|__|__|__|__|__|__|__|
Téléphone professionnel |__|__|__|__|__|__|__|__|__|__|
Adresse email _____________________@__________________

PARTIE A RENSEIGNER PAR LõENQUETEUR SEUL
UNE FOIS LA PASSATION DU QUESTIONNAIRE ACHEVEE

**U1. Durée de lõentretien**	minutes |____|____|____|

**U2. Lõenquêté(e)é**	Oui	Non
Comprenait mal le Français	1	2
A fait omissions volontaires sur sa vie	1	2

**U3. Lõentretien sõesté**	
Bien déroulé	1
moyennement passé	2
Mal passé	3

**U4. Dõautres personnes étaient-elles présentes susceptibles dõécouter lõinterview ?**	Oui	Non
conjoint	1	2
enfants	1	2
Autre : (précisez) é .	1	2

**U5. Compte tenu de la personne interrogée et du déroulement de lõinterview, diriez-vous que la qualité des données recueillies est**	
Très bonne	1
bonne	2
acceptable mais avec des réserves	3
douteuse	4

**U6. Y a-t-il des questions ou des sujets qui ont soulevé des problèmes ? Si oui, lesquels ?**

**U7. Dans quel type de lieu habite la personne interrogée ?**	
Une maison occupée par une ou deux familles	1
Une maison avec une ou deux familles mais ayant des parties communes avec une autre maison ou un autre immeuble (terrasse, jardin)	2
Un immeuble à un ou deux étages	3
Un immeuble de trois à cinq étages	4
Un immeuble de 6 à 10 étages	5
Un immeuble de plus de 10 étages	6

U8. Dans quel état sont les bâtiments et les habitations au voisinage du logement enquêté ?	
Très bon état	1
Bon état	2
Ni bon ni mauvais	3
Mauvais état	4
Très mauvais état	5

U9. Dans quel état est le bâtiment correspondant à lôadresse tirée au sort par rapport aux bâtiments et habitations alentours ?	
En bien meilleur état	1
En meilleur état	2
A peu près dans le même état	3
En plus mauvais état	4
En beaucoup plus mauvais état	5

U10. Avez-vous dôautres commentaires ?

Vérifiez une dernière fois que :

- **vous avez bien complété le <u>NumId</u> sur la couverture**
- **vous avez bien noté les <u>coordonnées</u> de la personne enquêtée**

Renvoyez les questionnaires par lot de 5 (jusquôà 1 kg) à lôadresse suivante :
Dr Pierre Chauvin, Inserm U707, 27 rue Chaligny, 75012 Paris

- **en indiquant votre nom <u>et INSERM U707</u> en expéditeur**

Pour tout renseignement,
vous pouvez joindre Barbara Schuch au 01 44 73 84 67 ou au 06 22 29 01 96
