# Supplementary material for: Who self-medicates? Results from structural equation modeling in the Greater Paris area, France
Source: PLoS One. 2018 Dec 17;13(12):e0208632. doi: 10.1371/journal.pone.0208632 (PMC6296538; doi:10.1371/journal.pone.0208632)
Supplement: S1 Fig — (PDF) [file pone.0208632.s002.pdf]

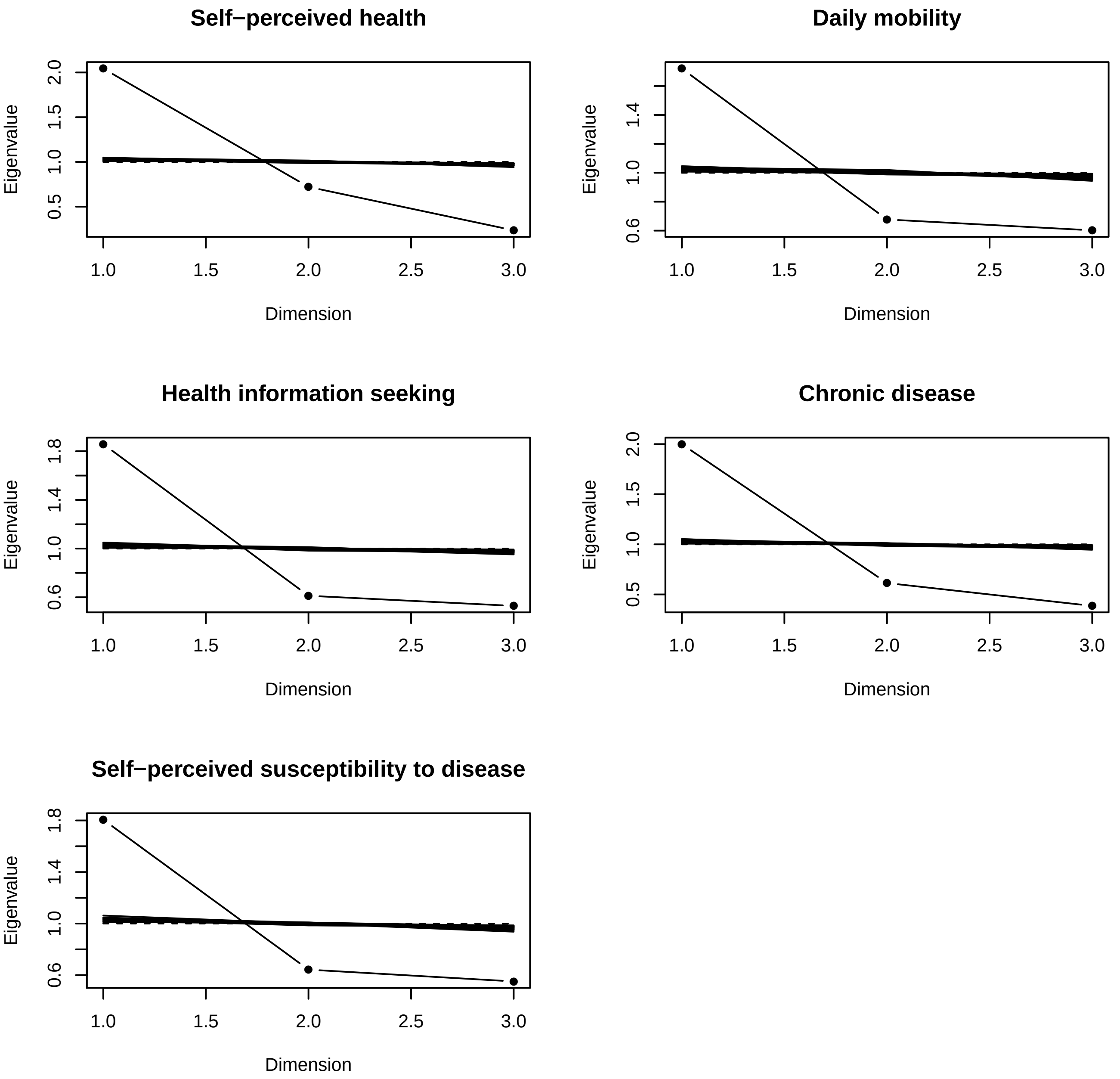

Self−perceived health
Daily mobility
Health information seeking
Chronic disease
Self−perceived susceptibility to disease
Eigenvalue
Dimension
0.5
1.0
1.5
2.0
0.6
1.4
1.8
2.5
3.0
