# Supplementary material for: Who self-medicates? Results from structural equation modeling in the Greater Paris area, France
Source: PLoS One. 2018 Dec 17;13(12):e0208632. doi: 10.1371/journal.pone.0208632 (PMC6296538; doi:10.1371/journal.pone.0208632)
Supplement: S2 Fig — N = 2918. Ellipses: latent variables; boxes: observed variables. All coefficients are standardized and have a p value <0.001. Robust CFI = 0.973. Robust RMSEA = 0.030. (PDF) [file pone.0208632.s003.pdf]

S2 Figure. Results of confirmatory factor analysis. N = 2918.

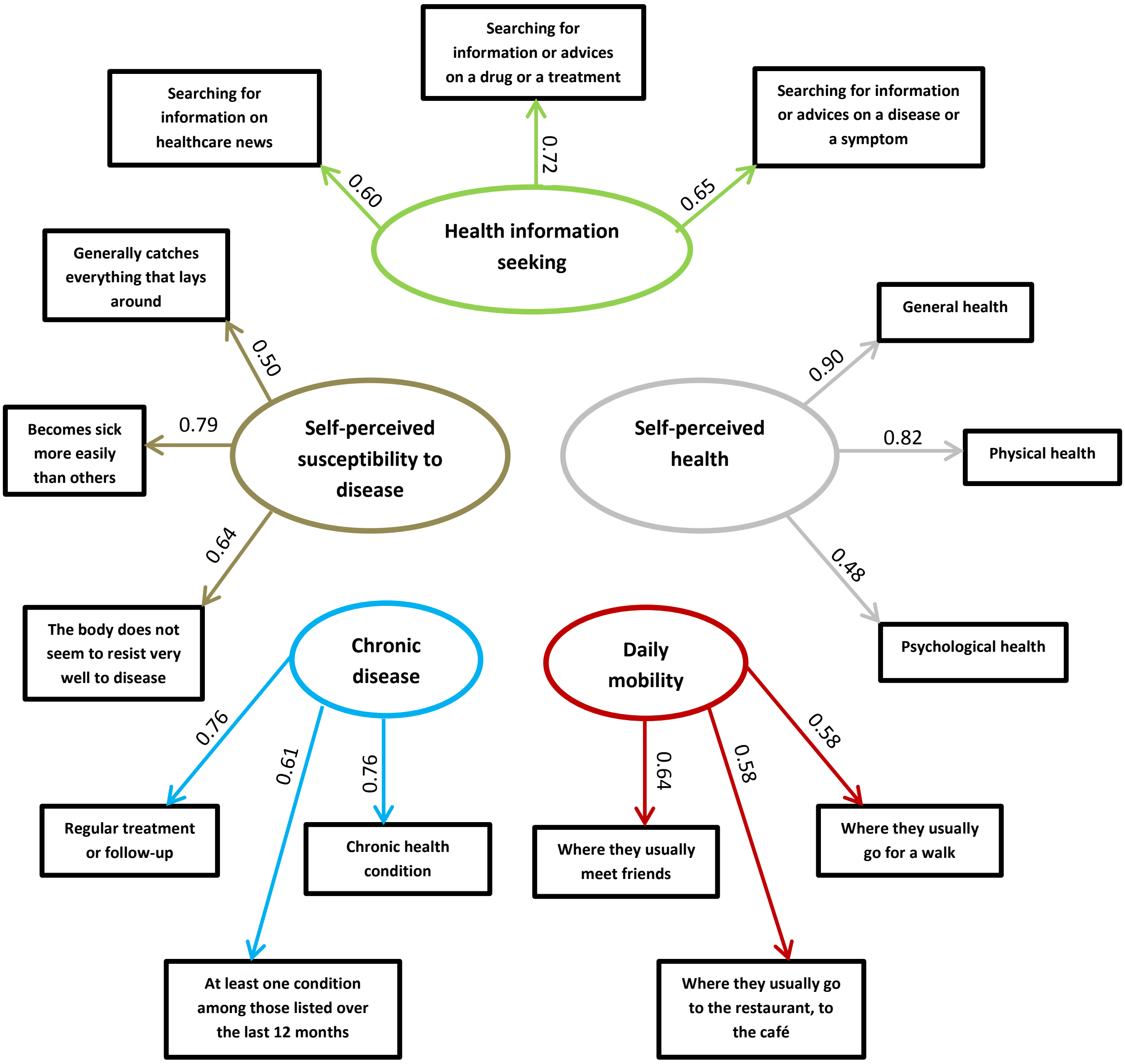

Ellipses: latent variables; boxes: observed variables. All coefficients are standardized and have a p value <0.001. Robust CFI = 0.973. Robust RMSEA = 0.030.
