# Supplementary material for: Who self-medicates? Results from structural equation modeling in the Greater Paris area, France
Source: PLoS One. 2018 Dec 17;13(12):e0208632. doi: 10.1371/journal.pone.0208632 (PMC6296538; doi:10.1371/journal.pone.0208632)
Supplement: S3 Fig — Arrows starting from the same explanatory variable are in the same colour. Ellipses: latent variables; boxes: observed variables. All coefficients are standardized. Robust CFI = 0.882. Robust RMSEA = 0.048. (PDF) [file pone.0208632.s004.pdf]

S3 Figure. Path diagram of self-medication in the bottom quartile of household income, n = 707.

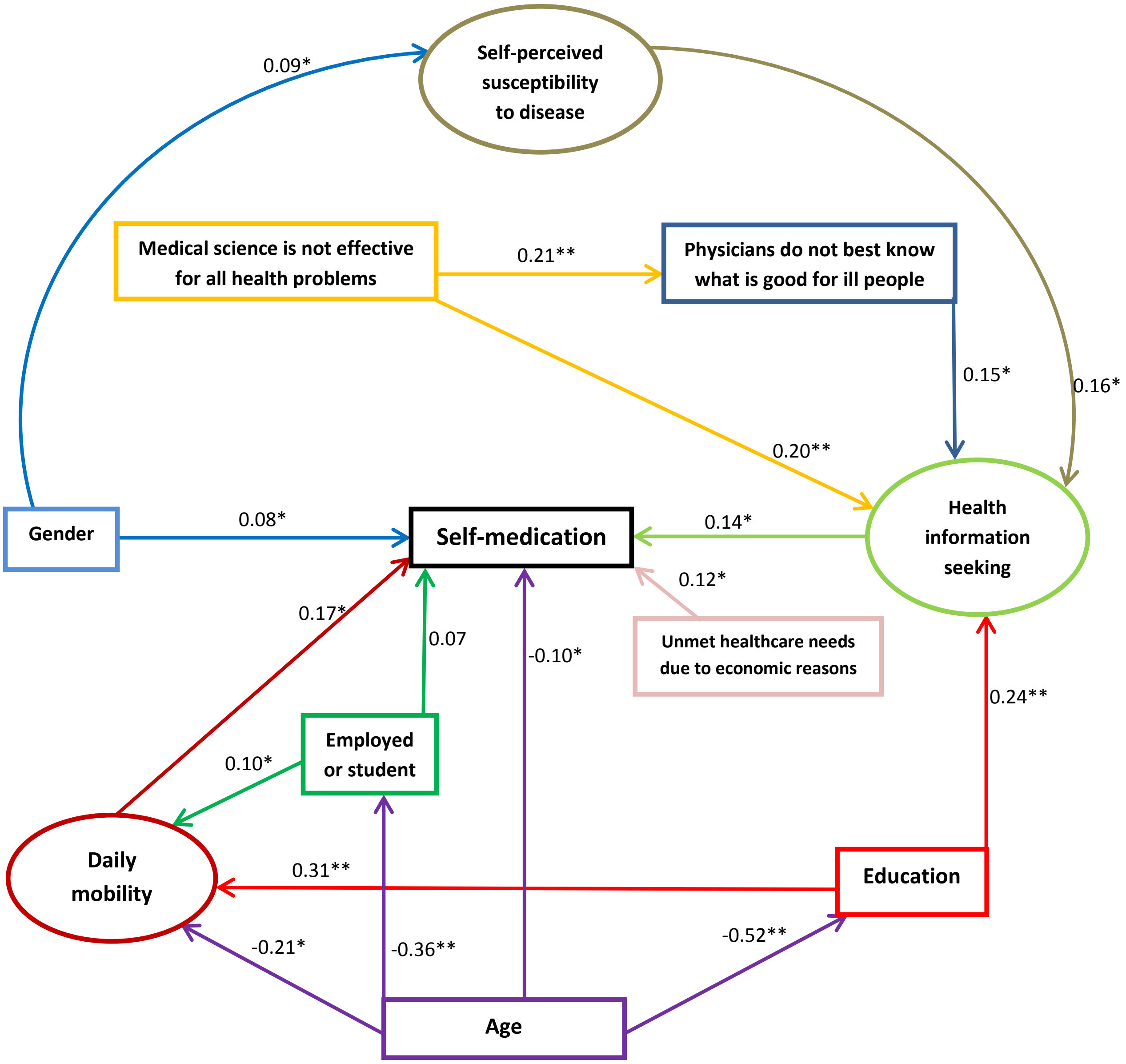

* : <0.05 ** : <0.001

Arrows starting from the same explanatory variable are in the same color. Ellipses: latent variables; boxes: observed variables. All coefficients are standardized. Robsut CFI = 0.882. Robust RMSEA = 0.048
