# Supplementary material for: Who self-medicates? Results from structural equation modeling in the Greater Paris area, France
Source: PLoS One. 2018 Dec 17;13(12):e0208632. doi: 10.1371/journal.pone.0208632 (PMC6296538; doi:10.1371/journal.pone.0208632)
Supplement: S4 Fig — Arrows starting from the same explanatory variable are in the same colour. Ellipses: latent variables; boxes: observed variables. All coefficients are standardized. Robust CFI = 0.946. Robust RMSEA = 0.030. (PDF) [file pone.0208632.s005.pdf]

S4 Figure. Path diagram of self-medication in the top three quartiles of household income, n = 2186

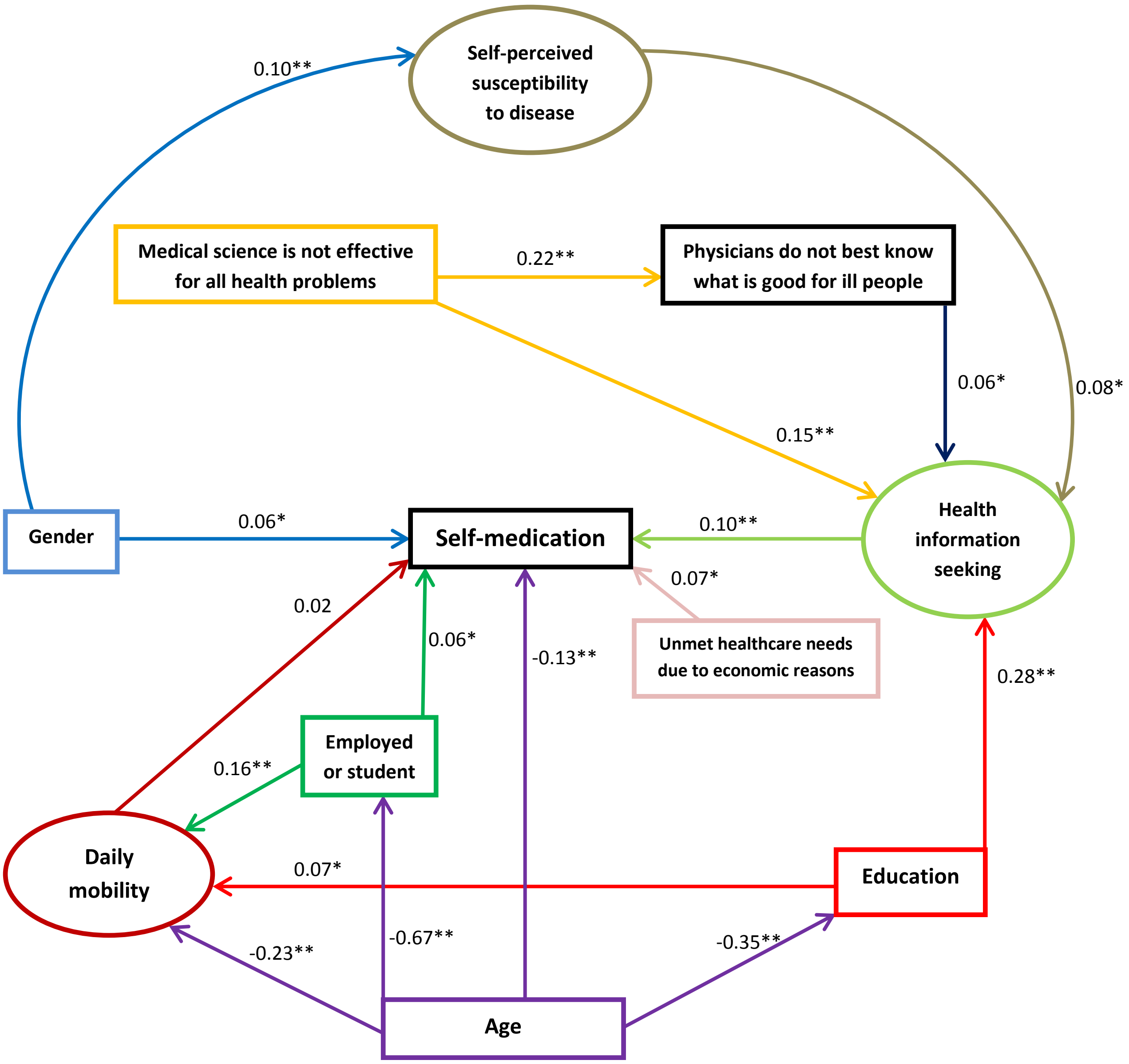

* : <0.05 ** : <0.001

Arrows starting from the same explanatory variable are in the same color. Ellipses: latent variables; boxes: observed variables. All coefficients are standardized. Robust CFI = 0.946. Robust RMSEA = 0.030.
